# Entangled photons enabled ultrafast stimulated Raman spectroscopy for molecular dynamics

**DOI:** 10.1038/s41377-024-01492-4

**Published:** 2024-07-15

**Authors:** Jiahao Joel Fan, Zhe-Yu Ou, Zhedong Zhang

**Affiliations:** 1 Department of Physics, City University of Hong Kong, Kowloon, Hong Kong SAR China; 2 City University of Hong Kong, Shenzhen Research Institute, Shenzhen, Guangdong China

**Keywords:** Quantum optics, Nonlinear optics, Ultrafast photonics

## Abstract

Quantum entanglement has emerged as a great resource for studying the interactions between molecules and radiation. We propose a new scheme of stimulated Raman scattering with entangled photons. A quantum ultrafast Raman spectroscopy is developed for condensed-phase molecules, to monitor the exciton populations and coherences. Analytic results are obtained, showing an entanglement-enabled time-frequency scale not attainable by classical light. The Raman signal presents an unprecedented selectivity of molecular correlation functions, as a result of the Hong-Ou-Mandel interference. Our work suggests a new paradigm of using an unconventional interferometer as part of spectroscopy, with the potential to unveil advanced information about complex materials.

## Abstract (web summary)

Proposed set up for entangled photons enabled ultrafast stimulated Raman spectroscopy for molecular dynamics.

## Graphical abstract

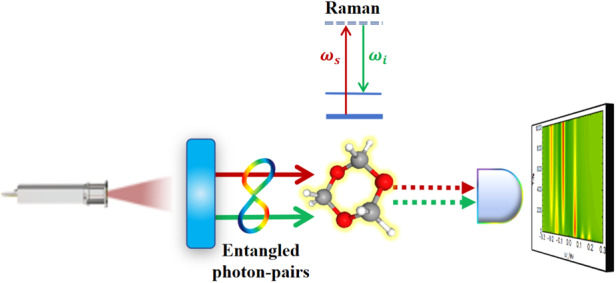

## Introduction

With the advancements of quantum light sources, the study of spectroscopy and sensing draw much attention in diverse fields of active research^1–8^. Quantum states of light with different types of entanglement offer new freedom for the light-matter interactions, spectroscopy and precise measurement^9–18^. Novel knobs can be developed thereby for controlling the atom and molecule motions at the microscopic scale. For instance, the capability of controlling the multi-photon transitions, as a signature of nonlinear optical processes, was enabled by photon entanglement^19–21^. Much attention have been drawn recently to the importance of entangled photons in various fields including quantum simulations^22^ and further the marriage of molecular spectroscopy with quantum photonics.

The multi-photon interactions with complex molecules were studied recently in a context of quantum-light spectroscopy. Several experiments indicated the extraordinary transitions with entangled two-photon absorption (ETPA)—the inhomogeneous line broadening can be circumvented for an efficient population of highly-excited states of molecules^1,5,7,8,19,23–32^. The multi-photon interaction has been studied much in atoms, it is however an open issue in molecules so far. This arises predominately from the couplings of electrons to more degrees of freedom, which brings up new challenges for the optical response. Elaborate experiments demonstrated the incredible power of entangled photons yielding the probe and control of electronic structures with unprecedented scales^33^. Recent studies extended the ETPA to a time-resolved regime, showing miraculous cancellation of molecular correlation functions not accessible by classical pulses^34^. New control knobs by entangled photons may enable considerable suppression of background in the radiation. An improvement in signal-to-noise ratio can be thus expected. The entanglement-refined interactions with molecules may induce a nonlinearity prominently resonant with the excited-state relaxation as well as the many-particle couplings. These call for a thorough understanding of the quantum-light interactions with complex molecules at ultrafast timescales.

The Raman process, as a typical component of the multi-photon interactions, closely connects to the quantum-light fields^35,36^. Extensive studies have demonstrated the quantum advantage of entangled light in spectroscopy^37–39^. The time-frequency entanglement of photons may enable a super-resolved capability, free of the conjugation of temporal and spectral scales that possess the fundamental limit in Raman spectroscopy using classical light. Recent work investigated the time-frequency entanglement in photon pairs before and after up-conversion^40^. In the context of these, our work highlights the unprecedented time-frequency scales by including the quantum-light interactions with molecules explicitly. This would be an important step toward spectroscopy and sensing, as they require the couplings to molecules which may lead to dramatic changes in the photon states.

Coherent Raman spectroscopy including a variety of schemes provides a powerful tool for quantum physics and materials characterization. A femtosecond coherent anti-Stokes Raman spectroscopy (CARS) was proposed recently with entangled photon pairs, to monitor the ultrafast dynamics of electronic coherence and the passage of conical intersections^41^. Furthermore, recent progress reported a CARS with squeezed photons in a nonlinear interferometer^42^. A quantum-enhanced measurement beyond the shot-noise limit was therefore performed. As a different scheme, the stimulated Raman scattering is sensitive to the molecular populations that are of fundamental interest and importance for the cooperative effects and multi-exciton correlations^43,44^. These can be monitored in a greater way by making use of entangled photons and nonlinear interferometry^45,46^.

In this article, we propose an ultrafast stimulated Raman spectroscopy (USRS) using entangled photons. A microscopic theory is developed with molecular trimers. Here the molecules play an active role as beam mixers for Raman pump and probe fields, rather than a passive role as beam splitters to scatter light only. Due to the entanglement, such a quantum USRS (Q-USRS) enables a super-resolved nature of the spectrum with time-frequency scales beyond the classical bound. Moreover, as a result of the multi-photon quantum interference, the spectroscopic signals are presented with an unprecedented selectivity, enabling selective access to molecular correlation functions. This is a hard task for classical pulses, but useful in spectroscopy^47^. Our work provides a new paradigm for Raman spectroscopy and metrology, insightful for the study of heterostructural materials.

## Results

### Theoretical framework

**Fig. 1:**
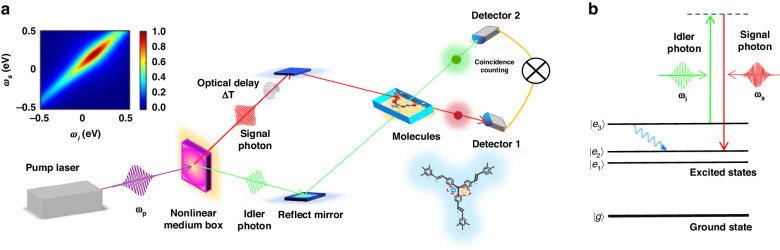
The schematic illustration of ultrafast stimulated Raman spectroscopy (USRS) with entangled photons. **a** Schematic of entangled twin photons (correlated with each other rather than the anti-correlation) as an ultrafast probe for molecules, where the nonlinear mediums and the photon-coincidence counting measurement are presented; Small panel plots ∣Φ(*ω*_*s*_, *ω*_*i*_)∣ of the entangled twin photons. **b** Level scheme of molecular relaxation interacting with two entangled photons that induce the stimulated Raman scattering

**Fig. 2:**
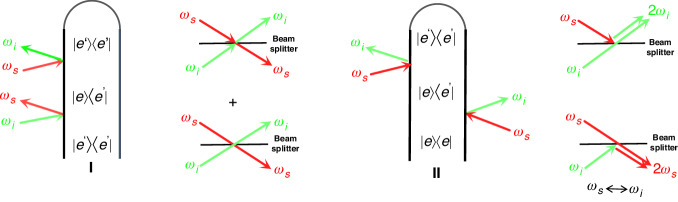
Feynman’s loop diagrams for the stimulated Raman scattering with twin photons. (I) Parametric process and (II) dissipative process. Two pathways of the two-photon interference in a fashion of the Hong–Ou–Mandel (HOM) scheme correspond to the processes (I) and (II). Notice that the complicated light–molecule interactions lead to the two-photon interference not exactly the same as the original HOM using 50:50 beam splitters

We consider a generic model of molecules interacting with entangled photons generated by nonlinear mediums in Fig. 1a. The two entangled photons are shaped in a short pulse. The photons in s and i arms are jointly scattered by molecular excited states, inducing the stimulated Raman process. A coincidence counting of emission is measured, where no spectrometers are required. The Raman interaction between molecules and entangled photons is of the form

1 $$V(t)=\alpha (t){E}_{s}(t){E}_{i}^{{\dagger} }(t)+\,{{\mbox{h.c.}}}$$

where *E*_*s*_(*t*) and *E*_*i*_(*t*) play the roles of the respective pump and probe fields containing multiple frequency modes. $\alpha (t)={\sum }_{m\ > \ n}{\alpha }_{mn}\left\vert {\psi }_{m}\right\rangle \left\langle {\psi }_{n}\right\vert (t)+\,{{\mbox{h.c.}}}$ defines the Raman polarizability operator and the elements *α*_*mn*_ are given in Supplementary Information (SI). Usually $\left\vert {\psi }_{m}\right\rangle \langle {\psi }_{n}| (t)=| {\psi }_{m}\rangle \left\langle {\psi }_{n}\right\vert {e}^{i{\omega }_{mn}t}$ for closed systems but we will not adopt this assumption, so as to involve more general cases described by a reduced density matrix. Equation (1) resembles the beam-splitter interaction, indicating the two-photon interference that essentially interplays with the molecular excitations. Further results will elaborate the active role of the Hong-Ou-Mandel (HOM) effect in Raman spectroscopy for the monitoring of excited-state dynamics. The Q-USRS is defined as the coincidence counting of the transmissions along s and i arms, yielding the signal $S({T}_{s},{T}_{i})=\langle {E}_{s}^{{\dagger} }(t){E}_{i}^{{\dagger} }(t){E}_{i}(t){E}_{s}(t)\rangle$, i.e.,

2 $$\begin{array}{lll}S({T}_{s},{T}_{i})=\Re \displaystyle{\iint}_{-\infty}^{+\infty }d{\omega }^{{\prime} }d\omega dtd\tau \,\theta (t-\tau )\\\times \left[\langle \psi (\tau )| \alpha (t-\tau )\alpha | \psi (\tau )\rangle {C}_{\rm{I}}(t,\tau ;{T}_{s},{T}_{i})\right. \\ \left.-\, \langle \psi (\tau )| \alpha \alpha (t-\tau )| \psi (\tau )\rangle {C}_{\rm{II}}(t,\tau ;{T}_{s},{T}_{i})\right]\end{array}$$

where *ψ*(*τ*) is the molecular wave function including full degrees of freedom. *T*_*s*_ and *T*_*i*_ are the central times of the pulses in s and i photons, respectively. Δ*T* = *T*_*i*_ − *T*_*s*_ measures the time delay between s and i photons that is controllable via optical paths. This provides the arrival times of the two photons with delays relative to the resonant pump that creates the electronic excitations in molecules, shown in Fig. 1b. The six-point field correlation functions are ${C}_{{{{\rm{I}}}}}(t,\tau ;{T}_{s},{T}_{i})={e}^{i\omega (t-{T}_{s})}{{{{\mathscr{F}}}}}_{{{{\rm{I}}}}}(t,\tau ;{T}_{s},{T}_{i})+(s\leftrightarrow i)$ and ${C}_{{{{\rm{II}}}}}(t,\tau ;{T}_{s},{T}_{i})={e}^{i\omega (t-{T}_{s})}{{{{\mathscr{F}}}}}_{{{{\rm{II}}}}}(t,\tau ;{T}_{s},{T}_{i})+(s\leftrightarrow i)$. Hereby, ${{{{\mathscr{F}}}}}_{{{{\rm{I}}}}}$ and ${{{{\mathscr{F}}}}}_{{{{\rm{II}}}}}$ are given by

3a $${{{{\mathscr{F}}}}}_{{{{\rm{I}}}}}=\langle \Psi | {{{{\mathscr{N}}}}}_{s}({\omega }^{{\prime} }){{{{\mathscr{E}}}}}_{i}^{{\dagger} }(\omega ){E}_{s}(t-{T}_{s}){E}_{s}^{{\dagger} }(\tau -{T}_{s}){E}_{i}(\tau -{T}_{i})| \Psi \rangle$$

3b $${{{{\mathscr{F}}}}}_{{{{\rm{II}}}}}=\langle \Psi | {E}_{s}^{{\dagger} }(\tau -{T}_{s}){E}_{i}(\tau -{T}_{i}){{{{\mathscr{N}}}}}_{s}({\omega }^{{\prime} }){{{{\mathscr{E}}}}}_{i}^{{\dagger} }(\omega ){E}_{s}(t-{T}_{s})| \Psi \rangle$$

where ${{{{\mathscr{N}}}}}_{s}({\omega }^{{\prime} })={{{{\mathscr{E}}}}}_{s}^{{\dagger} }({\omega }^{{\prime} }){{{{\mathscr{E}}}}}_{s}({\omega }^{{\prime} })$. The two components with *C*_I_ and *C*_II_ in Eq. (2) correspond to the loop diagrams in Fig. 2 which govern the multipoint Green’s functions of Raman operators. It includes the components: pathway I for the parametric process and pathway II for the dissipative process^48^. Notably from Eq. (2), in general,

4 $${C}_{{{{\rm{I}}}}}\,\ne\, {C}_{{{{\rm{II}}}}}\,\,{{\mbox{for quantum fields}}}\,;\quad {C}_{{{{\rm{I}}}}}={C}_{{{{\rm{II}}}}}\,\,{{\mbox{for classical fields}}}$$

One can probably achieve the selectivity of the molecular correlation functions, not attainable by classical pulses. The density matrix $\rho (\tau )=\left\vert \psi (\tau )\right\rangle \left\langle \psi (\tau )\right\vert$ contains full information about the structures and dynamics of molecules. These are essentially imprinted into the Raman signal, so as to be read out by making use of the quantum-field correlations.

### Q-USRS with entangled photons

To acquire the quantum-field correlations resulting from entangled photons, we consider the entangled state of photons in Fig. 1a is of the form $\left\vert \Psi \right\rangle ={\iint }_{-\infty }^{+\infty }\,{{\mbox{d}}}{\omega }_{s}{{\mbox{d}}}\,{\omega }_{i}\Phi ({\omega }_{s},{\omega }_{i}){a}_{{\omega }_{s}}^{{\dagger} }{a}_{{\omega }_{i}}^{{\dagger} }\left\vert 0\right\rangle$ and the two-photon wave function inside is read as

5 $$\Phi ({\omega }_{s},{\omega }_{i})=A({\omega }_{s}-{\omega }_{i}-{\omega }_{-})\phi \left[\frac{k({\omega }_{s},{\omega }_{i})L}{2}\right]{e}^{ik({\omega }_{s},{\omega }_{i})L/2}$$

with a phase matching $k({\omega }_{s},{\omega }_{i})L=\left({\omega }_{s}-\frac{{\omega }_{+}}{2}\right){\tau }_{s}+\left({\omega }_{i}-\frac{{\omega }_{+}}{2}\right){\tau }_{i}$, where *τ*_*s*_(*τ*_*i*_) is the time delay of s(i)-arm photons relative to the pump field *A*, due to the group velocity dispersion in the nonlinear mediums. *A* is a classical field with an effectively narrow bandwidth *σ*_0_, so that *A*(*ω*_*s*_ − *ω*_*i*_ − *ω*_−_) → *δ*(*ω*_*s*_ − *ω*_*i*_ − *ω*_−_) as *σ*_0_ → 0. *ω*_*s*_ − *ω*_*i*_ indicates quantum correlated photon pairs [the inset in Fig. 1a showing ∣Φ(*ω*_*s*_, *ω*_*i*_)∣] rather than the anti-correlated nature directly from the spontaneous down-conversion process.

Notably, the entangled photon states cause a miraculous cancellation of the field correlation functions. In particular, from Eqs. (3a), (3b) and (5),

6 $${C}_{{{{\rm{I}}}}}\,\ne\, 0,\quad {C}_{{{{\rm{II}}}}}=0$$

This is a typical two-photon interference, arising from the HOM effect^10^. As a result, the parametric component (loop diagram I) in Fig. 2 survives whereby the loop diagram II vanishes. A great selectivity of molecular Green’s functions is thus expected, not accessible by the Raman spectroscopy using classical pulses in which the loop diagrams I & II do survive [See Eq. (4)]. It should be aware that the Raman interaction in Eq. (1) largely differs from the 50:50 beam splitters in quantum optics. The destructive two-photon interference is therefore retained, giving *C*_I_ ≠ 0 rather than causing the full cancellation as in the original HOM scheme. The constructive two-photon interference groups two photons in one output mode, making *C*_II_ = 0. These yield an aspect of conceptual importance for quantum-light spectroscopy because the residue part characterizes the spectral lines that may provide a key monitoring of molecular structures and relaxation. Elaborate simulations of the Q-USRS incorporating the two-photon interference will be performed later on.

On substituting Eq. (5) into Eq. (2), we obtain the entangled Q-USRS, i.e.,

7 $$\begin{array}{l}S({\omega }_{-};{T}_{s},{T}_{i})\propto \displaystyle \mathop{\sum}\limits_{e,{e}^{{\prime}}}\mathop{\sum}\limits_{{e}^{{\prime\prime} }}\iint dtd\tau {e}^{-i{\omega }_{{e}^{{\prime\prime} }{e}^{{\prime} }}(t-\tau )}{\rho }_{e{e}^{{\prime} }}(\tau )\\\times\left[{\tilde{\Phi }}^{* }(t-{T}_{i},\tau -{T}_{i})\tilde{\Phi }(\tau -{T}_{s},t-{T}_{i})\right.\\\left.+\,{\tilde{\Phi }}^{* }(\tau -{T}_{s},t-{T}_{s})\tilde{\Phi }(t-{T}_{s},\tau -{T}_{i})\right]\end{array}$$

where $\tilde{\Phi }({t}_{1},{t}_{2})=\frac{1}{4{\pi }^{2}}{\iint }_{-\infty }^{+\infty }\Phi ({\omega }_{1},{\omega }_{2}){e}^{-i({\omega }_{1}{t}_{1}+{\omega }_{2}{t}_{2})}\,{{\mbox{d}}}{\omega }_{1}{{\mbox{d}}}\,{\omega }_{2}$ is the wave packet of the twin photons. The background has been dropped from Eq. (7), in that no spectral lines are produced. Eq. (7) indicates the role of the quantum-entangled photons whose unusual band properties may provide a versatile tool for controlling the fast excited-state dynamics of molecules.

Furthermore, we will proceed from Eq. (7) by assuming narrow-band field *A*, for some generic properties of the Q-USRS, i.e., time-frequency scales that highlight super-resolved nature. Defining $\tilde{\phi }(t)=\frac{1}{2\pi}\int\nolimits_{-\infty }^{+\infty }{e}^{-ivt}\phi (v)dv$ and assuming identical group velocity for s and idler photons, i.e., *τ*_*s*_ = *τ*_*i*_ = *τ*_0_, the twin photons present a pulse duration of *τ*_0_ (SI). The Q-USRS thus reads $S({\omega}_{-};{T}_{s},{T}_{i})=\Re \left[{{{\mathscr{S}}}}({\omega}_{-};{T}_{s},{T}_{i})\right]+(s\leftrightarrow i)$ with (${\omega}_{-}:= {\omega}_{-}+i{\sigma}_{0},{\omega}_{{e}^{\prime \prime}{e}^{{\prime}}}:= {\omega }_{{e}^{{\prime \prime}}{e}^{{\prime} }}-i{\gamma }_{{e}^{{\prime \prime} }{e}^{{\prime}}}$)

8 $$\begin{array}{l}{{{\mathscr{S}}}}({\omega}_{-};{T}_{s},{T}_{i})\approx \frac{{e}^{-\frac{i}{2}\varpi \Delta T}}{{(2\pi {\tau}_{0})}^{2}}\displaystyle \mathop{\sum}\limits_{e,{e}^{{\prime}}}\mathop{\sum}\limits_{{e}^{{\prime\prime}}}\frac{i}{{\omega}_{-}-{\omega}_{{e}^{{\prime\prime}}{e}^{{\prime} }}+\frac{{\omega}_{e{e}^{{\prime}}}}{2}}\\\displaystyle \times\int\nolimits_{-\infty}^{+\infty }dt\,{\rho }_{e{e}^{{\prime}}}(t)W\left(\frac{2t-2{T}_{i}-{\tau}_{0}}{{\tau}_{0}}\right)\end{array}$$

where *ϖ* = *ω*_−_ + *ω*_+_ and *W* is an overlapping function $W(\frac{t}{{\tau }_{0}})={\tilde{\phi }}^{* }(\frac{t}{{\tau }_{0}})\tilde{\phi }(\frac{t+\Delta T}{{\tau }_{0}})$, which indicates a decay of the destructive two-photon interference, when Δ*T* ≫ *τ*_0_. This evidences a promising HOM effect in the Q-USRS, as being sensitive to a simultaneous arrival of photons^49^. We observe from Eq. (8) a time window *T*_*i*_ ⩽ *t* ⩽ *T*_*i*_ + *τ*_0_ that extrapolates the molecular dynamics *ρ*(*t*). This yields real-time monitoring via a narrow window *τ*_0_ → 0. The spectral lines are given by the factor ${\omega }_{-}-{\omega }_{{e}^{{\prime\prime} }{e}^{{\prime} }}+\frac{{\omega }_{e{e}^{{\prime} }}}{2}$, subject to a variance *σ*_0_. Hence a time-frequency-resolved nature of the entangled Q-USRS is promising. To see this closely, we let Δ*T* = *T*_*i*_ − *T*_*s*_ = 0 and consider the population dynamics *ρ*_*ee*_(*t*) which dominates at a longer timescale. Equations (7) and (8) yield

9 $$S({\omega }_{-};{T}_{s},{T}_{s})\mathop{\propto}\limits_{ \sim }\frac{1}{{(2\pi {\tau }_{0})}^{2}}\mathop{\sum}\limits_{{e}^{{\prime} },e}\frac{{\sigma }_{0}+{\gamma }_{{e}^{{\prime} }e}}{{({\omega }_{-}-{\omega }_{{e}^{{\prime} }e})}^{2}+{({\sigma }_{0}+{\gamma }_{{e}^{{\prime} }e})}^{2}}\int\nolimits_{-\infty }^{+\infty }dt\,{\rho }_{ee}(t){\left\vert \tilde{\phi }\left(\frac{2t-2{T}_{s}-{\tau }_{0}}{{\tau }_{0}}\right)\right\vert }^{2}$$

The molecular dynamics is thus gated by a time window *T*_*s*_ ⩽ *t* ⩽ *T*_*s*_ + *τ*_0_. More advanced information about molecules and environments would be therefore unveiled. Elaborate results will be provided by simulating the Raman signal using certain molecular models.

### Molecular model

In the present work, we adopt the molecular aggregate model into the Q-USRS. The molecular Hamiltonian is given by

10 $${H}_{M}=\mathop{\sum }\limits_{n=1}^{N}{\omega }_{n}{\sigma }_{n}^{+}{\sigma }_{n}^{-}-J\mathop{\sum }\limits_{n=1}^{N-1}\left({\sigma }_{n+1}^{+}{\sigma }_{n}^{-}+\,{{\mbox{h.c.}}}\right)$$

where ${\sigma }_{n}^{+}$ is the raising operator of excitons at the *n*th molecule, in the limit of large onsite exciton-exciton coupling. *ω*_*n*_ is the exciton energy and *J* results from the dipole-dipole interaction between molecules. The model includes *N* photoactive molecules, so that a simple model for molecular trimer dyes can be obtained by *N* = 3. $V(t)={\sum }_{n = 1}^{N}{\sum }_{s}{f}_{ns}{\sigma }_{n}^{+}{\sigma }_{n}^{-}{Q}_{n,s}(t)$ gives the coupling of excitons to vibrations whereby *Q*_*n*,*s*_(*t*) is the coordinate of vibrations and *f*_*ns*_ is the coupling strength. Previous studies observed that the absorption/fluorescence spectrum of molecular aggregates shows a dense distribution of vibrational states attached to electronic excitations, evident by the inhomogeneous line broadening characterized by a smooth spectral density of vibrations^50,51^. Averaging over the vibrations and radiative loss, i.e., $\rho (t)={{{\mbox{Tr}}}}_{B}\left\vert \psi (t)\right\rangle \left\langle \psi (t)\right\vert$, the nonradiative relaxation is opened for the Frenkel excitons in the aggregates. We obtain the equation of motion $\dot{\rho }(t)=-i[{H}_{0},\rho (t)]+\hat{\,{{\mbox{W}}}\,}\rho (t)$ with the operator $\hat{\,{{\mbox{W}}}\,}$ that contains random jumps between the eigenstates of *H*_0_, i.e.,

11 $$\hat{\,{{\mbox{W}}}\,}\odot =\displaystyle\mathop{\sum}_{j > i}\frac{{\gamma }_{ji}}{2}\left[{\bar{n}}_{{\omega }_{ji}}\left({L}_{ji}^{+}\odot {L}_{ij}^{-}-\odot {L}_{ij}^{-}{L}_{ji}^{+}\right)+\left({\bar{n}}_{{\omega }_{ji}}+1\right)\left({L}_{ij}^{-}\odot {L}_{ji}^{+}-\odot {L}_{ji}^{+}{L}_{ij}^{-}\right)\right]+\,{{\mbox{h.c.}}}$$

where ⊙ denotes an operator. *γ*_*ji*_ ∝ *D*(*ω*_*ji*_) and *D*(*ω*_*ji*_) is the spectral density of vibrations^52^. ${\bar{n}}_{{\omega }_{ji}}$ is the thermal distribution of vibrations at frequency *ω*_*ji*_^53^. ${L}_{ji}^{+}=\mathop{\sum }\nolimits_{n = 1}^{N}{{{{\bf{P}}}}}_{jn}^{-1}{{{{\bf{P}}}}}_{ni}\vert {\psi }_{j}\rangle\langle {\psi }_{i}\vert$ and ${L}_{ij}^{-}={({L}_{ji}^{+})}^{{\dagger} }$ are the jump operators between Frenkel exciton states $\left\vert {\psi }_{i}\right\rangle$. Notice that $\{\left\vert {\psi }_{i}\right\rangle \}$ can include any number of excitons. The density-matrix dynamics, therefore, provide a microscopic description for the Q-USRS.

### Simulation of Q-USRS

**Fig. 3:**
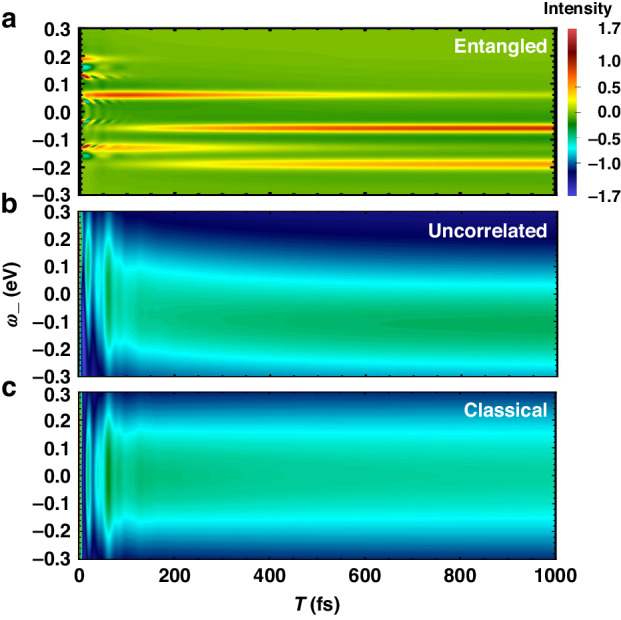
Signal of ultrafast stimulated Raman spectroscopy with different photon statistics. **a** Q-USRS signal with entangled photons from Eq. (7); **b** Q-USRS signal with uncorrelated photons; **c** FSRS signal using classical pulses. Parameters are: *σ*_0_ = 1 meV, *τ*_0_ = 25 fs, *ω*_+_ = 0.3 eV for (**a**), and ${\tilde{\sigma }}_{0}^{-1}=2{\tau }_{0}=50$ fs for (**b**, **c**); *ω*_1_ = 2.25 eV, *ω*_2_ = *ω*_3_ = 2.1 eV, *J* = 30 meV from PBI trimers^61^

**Fig. 4:**
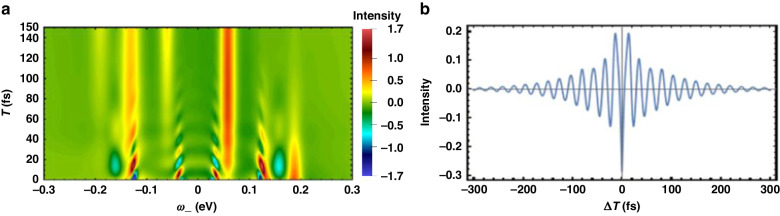
Signal of quantum ultrafast stimulated Raman spectroscopy with entangled photons, at shorter timescale. **a** Zoom-in of Fig. 3a for a short timescale; **b** Q-USRS signal with entangled photons varies with the optical delay ${\Delta}{T}\,=\,{T_{i}-T_{s}}$, at the Raman peak ${\omega }_{-}={\omega }_{{e}_{3},{e}_{2}}=0.13$ eV. Other parameters are the same as Fig. 3a

To gain an intuitive understanding of how entangled light influences stimulated Raman spectra in molecules, we use a Photosystem (PS) trimer model to simulate the Q-USRS with entangled photon pairs combined with a nonlinear interferometer scheme. The calculations are based on Eq. (7). Figure 3 shows the USRS signal with various photon statistics. The plots vary with *ω*_−_ and arrival time *T* of the two photons with a zero optical delay (Δ*T* = 0). Using the parameters from PS trimers, we consider *N* = 3 molecules and weak coupling *J* = 30 meV that yields small energy splitting as illustrated in Fig. 1b. Figure 3a displays several peaks presenting dramatically different behaviors when varying the delay *T* (arrival time of entangled photons). In particular, at the Raman shift of *ω*_−_ = −0.059, −0.189 eV, two long stripe-shaped peaks can be observed promisingly at *T* > 400 fs. The time-evolving dynamics ${\rho }_{{e}_{1},{e}_{1}}(T+\frac{{\tau }_{0}}{2})$ is thus monitored, meaning a downhill transfer of exciton population towards $\left\vert {e}_{1}\right\rangle$. Likewise, the peak intensity at *ω*_−_ = − 0.13, 0.059 eV shows a rapid increase during 100 fs and drops smoothly therein. This monitors the dynamics of exciton population at $\left\vert {e}_{2}\right\rangle$, i.e., ${\rho }_{{e}_{2},{e}_{2}}(T+\frac{{\tau }_{0}}{2})$. The two peaks at *ω*_−_ = 0.13, 0.189 eV resolve the exciton dynamics at $\left\vert {e}_{3}\right\rangle$, whose population drops dramatically within 700 fs. Nevertheless, a few side peaks can be seen within a shorter timescale, by a zoom-in. The oscillatory nature evidences the quantum coherence of excitons, as detailed next. Figure 4a illustrates the fast dynamics of coherences coexisting with the exciton populations. The side peaks essentially monitor the coherences, provided that ${\rho }_{e{e}^{{\prime} }}(e\ne {e}^{{\prime} })$ are associated with the Raman resonances distinct from *ρ*_*ee*_, as given by Eq. (8). For instance, the peaks at *ω*_−_ = ± 0.124 eV resolve the excitonic coherence ${\rho }_{{e}_{2},{e}_{3}}$ and ${\rho }_{{e}_{3},{e}_{2}}$, when observing an oscillation period of 25 fs.

Indeed, the exciton dynamics is subject to a temporal scale *τ*_0_, whereas the line broadening of the Raman signal is given by *σ*_0_. These are evident by Eq. (8) and are confirmed from the simulations.

Figure 3b and c plot the USRS without quantum entanglement. The result using uncorrelated twin photons is given in Fig. 3b, where the each photon has a bandwidth ${\tilde{\sigma }}_{0}=\frac{1}{2{\tau }_{0}}$. A similar consideration applies to the classical USRS, whereby using two laser pulses with a bandwidth ${\tilde{\sigma }}_{0}=\frac{1}{2{\tau }_{0}}$ to drive the stimulated Raman process. Figure 3b, c evidence fairly blurred spectral lines and dynamics if the pulses are short. And the classical USRS dilutes the selectivity of molecular correlation functions, disclosing an intrinsic limit for accessing the excited-state dynamics. This can be seen from the symmetric distribution of the Stokes and anti-Stokes lines in Fig. 3c. Such a lack of pathway selectivity holds as well for the femtosecond stimulated Raman scattering (FSRS) using combined narrow- and broad-band pulses.

### Multi-photon interference

To see closely the quantum-light-enabled selectivity, we scan the optical delay Δ*T* = *T*_*i*_ − *T*_*s*_ so that the *s* and *i* photons have different arrival times. Figure 4b shows a slice of the entangled Q-USRS at ${\omega }_{-}={\omega }_{{e}_{3},{e}_{2}},T=0$. This is a typical HOM interference: a decaying envelope as the two photons get separated in time. The destructive interference is however retained, as a result of light-molecule interactions which yield a residue of the HOM dip at Δ*T* = 0. The dip intensity does not only reveal an overlap between wavepackets of twin photons but also imprints the electronic structure and dynamics of molecules. The latter is extensively important for spectroscopy and sensing, in that the dip intensity indeed engraves the interference between the two parametric processes given in Fig. 2. The photon pair at outgoing ports interferes with its interchange *s* ↔ *i* essentially.

Moreover, Fig. 4b illustrates a quantum beating with a dominant frequency of ∣*ω*_+_ + *ω*_−_∣/2 for the anti-Stokes Raman peaks and of ∣*ω*_+_ − ∣*ω*_−_∣∣/2 for Stokes Raman peaks. This makes sense once noting the frequency offset between the two wavepackets of the twin photons^49^.

## Discussion

In contrast to the experimental demonstrations so far, which mostly employed the frequency anti-correlated twin photons generated from the spontaneous parametric processes, the photon source required for our study is not easily available. However, the experimental methods were developed recently to engineer the spectral correlation by certain design^54–56^. In particular, the spectral correlation property required in our scheme, i.e., frequency positively correlated twin photons, has been achieved in experiments^55^, thus paving the road for the Raman spectroscopy application. The basic strategy was a clever integration of nonlinear interference scheme and phase-control device, in which the former was made by a group of nonlinear mediums with the parametric down conversion involved^57,58^. The spectral function of the photon pairs can be further modulated by the phase-control devices, leading to the programmable spectral properties of the photon pairs^55^. Further experimental endeavor on the Raman spectroscopy with the entangled photons is under way and will be presented in near future.

There were several quantum-optical experiments which had demonstrated the independent time and frequency scales in the entangled photons^54,55^. These experimental observations—by presenting a variety of advantages and superiority through a diversity of systems including atoms and qubits—are intimately connected to the Q-USRS. In ref. ^54^, it has been demonstrated for the time-frequency scale *δωδt* ≈ 10^−6^ using the entangled photons, through the pulse shaping technique that can have a precise control of temporal properties. The entangled photons have been further used for studying the coherent dynamics of magnons^21,59,60^, in which the two-photon interference played an important role resembling the Raman interaction in Eq. (1). Despite these achievements which indicate the time-frequency scales beyond the classical Fourier limitation, the interactions between entangled photons and complex molecules still remain elusive. This is a key step in the journey towards the quantum-light Raman spectroscopy.

In summary, (i) we develop a microscopic theory for the USRS with quantum-light fields, incorporating photon-coincidence counting. (ii) A simple analytic expression is derived for the Q-USRS using entangled photons, yielding elaborate HOM interference which enables the selective access of molecular correlation functions. (iii) Unprecedented time-frequency-resolved property of the Q-USRS is shown explicitly, from the fact that the temporal and spectral scales are not conjugated due to the quantum correlations of photons. (iv) Fast exciton dynamics are visualized including the fluctuating energy gap and populations in the real-time domain. (v) No gratings are needed for spectrally resolved lines in our investigated scheme.

Overall, all these yield unprecedented scales for spectroscopy to be facilitated with quantum advantage and would be sufficient for the tomography of the density matrix of molecules. Our work, as a new coherent Raman technique, may open a new frontier for studying the ultrafast processes in photochemistry, nano-plasmonics, and semiconducting heterostructures.

## Materials and methods

### Photon pairs

As illustrated in Fig. 1a, the entangled photon pairs allow us to employ the two-photon wave function in Eq. (5) as

12a $$A({\omega }_{s}-{\omega }_{i}-{\omega }_{-})=\frac{{\sigma }_{0}}{{({\omega }_{s}-{\omega }_{i}-{\omega }_{-})}^{2}+{\sigma }_{0}^{2}}$$

12b $$\phi \left[\frac{k({\omega }_{s},{\omega }_{i})L}{2}\right]=\frac{\frac{2i}{{\tau }_{0}}}{{\omega }_{s}+{\omega }_{i}-{\omega }_{+}+i\frac{2}{{\tau }_{0}}}$$

assuming *τ*_*s*_ = *τ*_*i*_ = *τ*_0_ along with before. In what follows, we assert that *ω*_−_ can be tuned whereas fixing *ω*_+_.

With the above consideration of utilizing entangled photon pair, we considered the scenario of uncorrelated photon pairs as well, in which two-photon wave function split to two independent photon wave functions as Φ(*ω*_*s*_, *ω*_*i*_) = *A*(*ω*_*s*_ − *ω*_*s*0_)*A*(*ω*_*i*_ − *ω*_*i*0_)

13 $$A(\omega -{\omega }_{0})=\frac{{\sigma }_{0}}{{(\omega -{\omega }_{0})}^{2}+{\sigma }_{0}^{2}}$$

where *ω*_0_ represents central frequency of each photon wave. When we consider classical Raman spectroscopy, joint detection is removed and the grating has to be taken into the pathway of each pulse, thus the six-point field correlation functions in Eq. (2) are reduced to the product of four corresponding classical fields

14a $${C}_{{{{\rm{I}}}}}={\varepsilon }_{i}^{{\dagger} }(t-{T}_{i}){\varepsilon }_{s}(t-{T}_{s}){\varepsilon }_{s}^{{\dagger} }(\tau -{T}_{s}){\varepsilon }_{i}(\tau -{T}_{i})+(s\leftrightarrow i)$$

14b $${C}_{{{{\rm{II}}}}}={\varepsilon }_{s}^{{\dagger} }(\tau -{T}_{s}){\varepsilon }_{i}(\tau -{T}_{i}){\varepsilon }_{i}^{{\dagger} }(t-{T}_{i}){\varepsilon }_{s}(t-{T}_{s})+(s\leftrightarrow i)$$

where ${\varepsilon }_{s/i}(t)={e}^{i({\omega }_{s/i}+i{\sigma }_{0})t}$. Note, the FSRS is hence a consequence of the summation of four SRS processes as demonstrated in SI.

### Microscopic theory

To illustrate the molecular dynamics, the density matrix *ρ* requires us to find the effective Hamiltonian of the molecular aggregate model. Without any generality, a *N* × *N* matrix of the free Hamiltonian can be diagonalized as ${H}_{0}^{D}={{{{\bf{P}}}}}^{-1}{H}_{0}{{{\bf{P}}}}$ with an invertible modal matrix **P**, which consists of eigenvectors of *H*_0_

15 $${H}_{0}^{D}={{{{\bf{P}}}}}^{-1}\left(\begin{array}{ccc}{\omega }_{1}&-J&0\\ -J&{\omega }_{2}&-J\\ 0&-J&{\omega }_{3}\end{array}\right){{{\bf{P}}}}=\left(\begin{array}{ccc}\widetilde{{\omega }_{1}}&0&0\\ 0&\widetilde{{\omega }_{2}}&0\\ 0&0&\widetilde{{\omega }_{3}}\end{array}\right)$$

Note, the parameters of molecular Hamiltonian are based on PS trimer system from the simulation result in ref. ^61^: *ω*_1_ = 2.25 eV,*ω*_2_ = *ω*_3_ = 2.1 eV, *J* = 30 meV. Having the diagonalized matrix ${H}_{0}^{D}$, the trimer system is transformed to an effective three-level system, which contains three new eigenstates $\left\vert {e}_{n}\right\rangle$ as shown in Fig. 1, and the corresponding eigenvalues $\widetilde{{\omega }_{n}}$ are: $\widetilde{{\omega }_{1}}\approx 2.25$ eV, $\widetilde{{\omega }_{2}}\approx 2.13$ eV, and $\widetilde{{\omega }_{3}}\approx 2.07$ eV. The extracted invertible modal matrix **P** is thus used to construct operator $\hat{\,{{\mbox{W}}}\,}$ in Eq. (11). Furthermore, we can rewrite the equation of motion $\dot{\rho }(t)=-i[{H}_{0},\rho (t)]+\hat{\,{{\mbox{W}}}\,}\rho (t)$ into the Liouville-space form

16 $$\dot{\rho }(t)={\mathbb{L}}\rho (t)$$

the matrix of Liouville operator ${\mathbb{L}}$ contains the evolutionary information about density matrix *ρ*(*t*), which can provide molecular dynamics to Q-USRS in Eq. (8)

### Numerical simulations

By running the integration of Eq. (8), the signals are presented in the form of a 2D spectrum. In addition to the main result as shown above, all relevant analytical and numerical results are provided in detail in SI.

### Supplementary information

Supplementary Information
