# Supplementary material for: Entangled photons enabled ultrafast stimulated Raman spectroscopy for molecular dynamics
Source: Light Sci Appl. 2024 Jul 15;13:163. doi: 10.1038/s41377-024-01492-4 (PMC11247098; doi:10.1038/s41377-024-01492-4)
Supplement: Supplementary file 1 — Supplementary Information [file 41377_2024_1492_MOESM1_ESM.pdf]

Supplementary Information for "Entangled Photons Enabled Ultrafast Stimulated Raman Spectroscopy for Molecular Dynamics"

JIAHAO JOEL FAN[1], ZHE-YU OU[1,*], AND ZHEDONG ZHANG[1,2,*]

[1] Department of Physics, City University of Hong Kong, Kowloon, Hong Kong SAR, China
[2] City University of Hong Kong, Shenzhen Research Institute, Shenzhen, Guangdong 518057, China
[*] jeffou@cityu.edu.hk
[*] zzhan26@cityu.edu.hk

1. INTRODUCTION

The math details in addition to the conclusions delivered in the main text have been provided. Meanwhile, some supplementary results on quantum Raman spectroscopy are shown, in support of the main text. In this document, we sophisticate the additional math derivation and carry out the calculation of the coincidence counting detection signal of stimulated Raman scattering with two entangled photons, uncorrelated photons, and a conventional detection signal with two classic pulses.

2. ULTRAFAST STIMULATED RAMAN SCATTERING PROCESS

In the present study, the ultrafast stimulated Raman scattering process totally consists of four transition pathways, and the main text has demonstrated two of them. Hence, in this material, we list all possible transition pathways of Raman scattering as well as corresponding Hong Ou Mandel configurations in Fig.S1. Fig.S3 has demonstrated that transition pathways I and III owe to the parametric processes, while transition pathways II and IV belong to the dissipative processes. By comparing the double-sided Feynman diagrams of USRS process with Hong Ou Mandel configurations with a 50/50 beam splitter, the parametric processes(I and III) exit two photons on each side, the dissipative processes are with respect to the Hong Ou Mandel configurations which allow two photons exit together on one side. Note, the parametric process which exits two photons on each side actually includes two equally probable possibilities: the incident twin photons both transmit through the sample molecules with energy conversion ($\omega_{s/i} \longleftrightarrow \omega_{i/s}$); the incident twin photons are both reflected in the sample molecules without energy conversion, as

demonstrated in FigS2.

A. Raman polarizability Operator

As mentioned in the main text, $\alpha(t) = \sum_{m>n} \alpha_{mn} |\psi_m\rangle\langle\psi_n|(t) + \text{h.c.}$ defines the Raman polarizability operator. Usually $|\psi_m\rangle\langle\psi_n|(t) = |\psi_m\rangle\langle\psi_n| e^{\mathbf{i}\omega_{mn}t}$ for closed systems but we will not adopt this assumption hereafter, in order to involve more general cases whose dynamics may be described by a reduced density matrix. The matrix element α_{mn} for excited states reads

$$\alpha_{mn} = \sum_k \frac{\mu_{mk}\mu_{kn}}{\hbar} \left(\frac{1}{\omega_k - \omega_n - \omega_s} + \frac{1}{\omega_k - \omega_m + \omega_i} \right) \tag{S1}$$

here, $\mu_{\alpha\beta}$ is the transition dipole between two electronic states. $\sum_k$ denotes the sum over virtual electronic states.

B. Two-photon wave function

The two-photon wave function employed in the main text is given

$$\begin{aligned}\Phi(\omega_s, \omega_i) =& A(\omega_s - \omega_i - \omega_-)\phi \left[\frac{k(\omega_s, \omega_i)L}{2} \right] e^{ik(\omega_s,\omega_i)L/2} \\ =& \frac{\sigma_0}{(\omega_s - \omega_i - \omega_-)^2 + \sigma_0^2} \frac{\mathbf{i}\frac{2}{\tau_0}}{\omega_s + \omega_i - \omega_+ + \mathbf{i}\frac{2}{\tau_0}} e^{\mathbf{i}(\omega_s+\omega_i-\omega_+)\tau_0/2}\end{aligned}$$

with ω_- and ω_+ are tuneable frequencies of the pump laser with respect to frequency correlation and anticorrelation types of the entanglement.

Since we transformed the two-photon wave function into the time domain in Eq. (4a) of the main text with $\tilde{\Phi}(t, t') = \frac{1}{4\pi^2} \iint_{-\infty}^{+\infty} \Phi(\omega_s, \omega_i) e^{-\mathbf{i}(\omega_1 t + \omega_2 t')} \mathrm{d}\omega_s \mathrm{d}\omega_i$ and assuming A is a classical field with an effectively narrow bandwidth σ_0, so that $A(\omega_s - \omega_i - \omega_-) \to \delta(\omega_s - \omega_i - \omega_-)$ as $\sigma_0 \to 0$. We applied the identical group velocity for s and idler photons, i.e., $\tau_s = \tau_i = \tau_0$ as well. Hence, the two-photon wavepacket is read as

$$\tilde{\Phi}(t, t') = c(t, t')\tilde{\phi}[(t + t' - \tau_0)/\tau_0] \tag{S2}$$

where $\tilde{\phi}(t) = \frac{1}{2\pi} \int_{-\infty}^{+\infty} e^{-ivt}\phi(v)dv$ and $c(t, t') = \frac{1}{2\pi\tau_0} e^{\mathbf{i}(\omega_- - \omega_+)(t'+t)/2} e^{-\mathbf{i}\omega_- t}$

Apart from the expressions of S_I and S_{II} shown in the main text, obtaining the corresponding double-sided Feynman diagrams of USRS transition pathways III and IV, the coincidence counting signals of two additional transition pathways III and IV are thus given by

$$\begin{aligned}S_{III}(T_s, T_i) =& \Re \iint_{-\infty}^{+\infty} dtd\tau\, \theta(t - \tau)\langle\psi(\tau)|\alpha(t - \tau)\alpha|\psi(\tau)\rangle C_{\mathrm{III}}(t, \tau; T_s, T_i) \\ =& \Re \iiiint_{-\infty}^{+\infty} d\omega' d\omega dt d\tau\, e^{i\omega'(t-T_i)} \langle\langle 1|\alpha_L G(t - \tau)\alpha_L|\rho(\tau)\rangle\rangle \\ & \times \langle\Psi|\mathcal{N}_i(\omega)\mathcal{E}_s^\dagger(\omega')E_i(t - T_i)E_i^\dagger(\tau - T_i)E_s(\tau - T_s)|\Psi\rangle\end{aligned} \tag{S3}$$

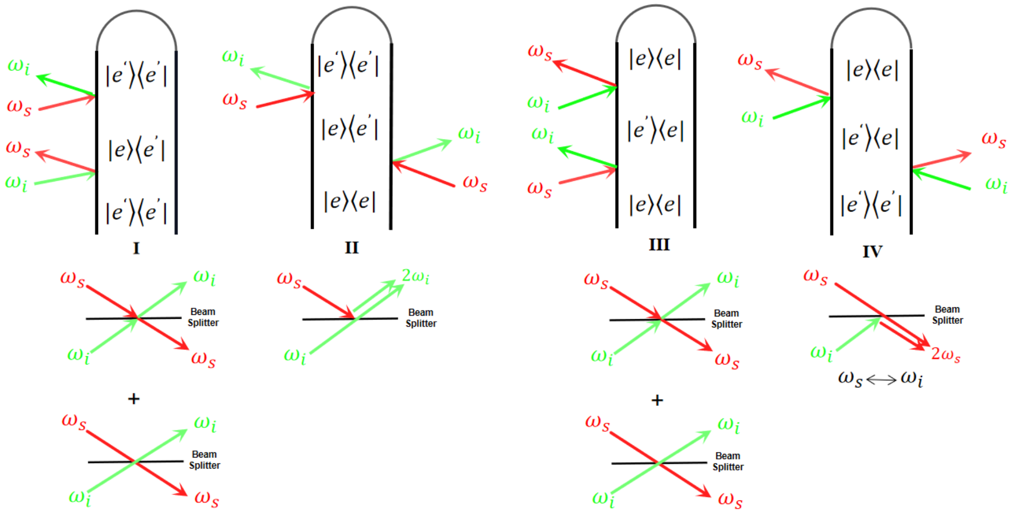

Fig. S1. Double-sided Feynman diagrams of USRS transition pathways and corresponding Hong Ou Mandel configurations.

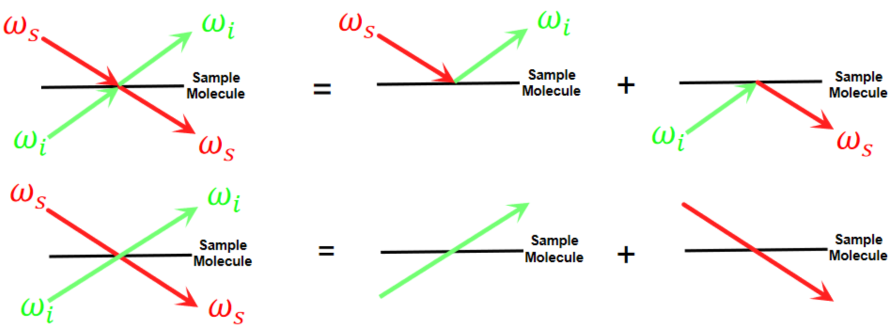

Fig. S2. The two possibilities of Hong Ou Mandel configurations I and III for parametric processes of USRS.

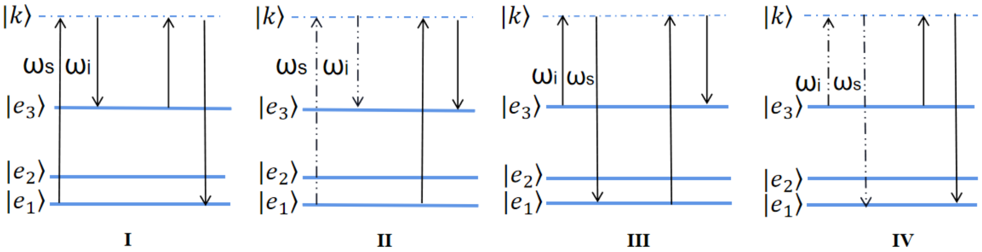

Fig. S3. Energy level diagrams of four possible USRS transition pathways, in which transition pathways I and III owe to the parametric processes, while transition pathways II and IV belong to the dissipative processes

$$
\begin{aligned}
S_{IV}(T_s,T_i) =& \Re \iint_{-\infty}^{+\infty} dtd\tau\ \theta(t-\tau)\langle\psi(\tau)|\alpha\alpha(t-\tau)|\psi(\tau)\rangle C_{\text{IV}}(t,\tau;T_s,T_i) \\
=& \Re \iiiint_{-\infty}^{+\infty} d\omega' d\omega dtd\tau\ e^{i\omega'(t-T_i)} \langle\langle 1|\alpha_L G(t-\tau)\alpha_R|\rho(\tau)\rangle\rangle \\
& \times \langle\Psi|E_i^\dagger(\tau-T_i)E_s(\tau-T_s)\mathcal{N}_i(\omega)\mathcal{E}_s^\dagger(\omega')E_i(t-T_i)|\Psi\rangle
\end{aligned}
\tag{S4}
$$

Herein, $\mathcal{F}_{\text{III}}(t,\tau;T_s,T_i) = \langle\Psi|\mathcal{N}_i(\omega)\mathcal{E}_s^\dagger(\omega')E_i(t-T_i)E_i^\dagger(\tau-T_i)E_s(\tau-T_s)|\Psi\rangle$ and $\mathcal{F}_{\text{IV}}(t,\tau;T_s,T_i) = \langle\Psi|E_i^\dagger(\tau-T_i)E_s(\tau-T_s)\mathcal{N}_i(\omega)\mathcal{E}_s^\dagger(\omega')E_i(t-T_i)|\Psi\rangle$ with $\mathcal{N}_i(\omega) = \mathcal{E}_i^\dagger(\omega)\mathcal{E}_i(\omega)$extracted from Eq.2 in the main text. As mentioned in the main text, transition pathways I and III owe to the parametric processes, while transition pathways II and IV belong to the dissipative processes, in which the two corresponding 6th-order correlation functions of coincidence counting signal $C_{\text{II}} = C_{\text{IV}} = 0$ and vanish in the total signal. Hence, the zero-contribution of the dissipative process simplifies the calculation of the coincidence counting signal and encourages us to focus on two parametric processes of USRS, namely S_I and S_{III}.

3. Q-USRS COINCIDENCE COUNTING SIGNAL WITH ENTANGLED PHOTONS

In this section, we carried out the calculation of S_I and S_{III}, both of which consist of spectral and background signals, furthermore, either spectral signal or background signal has both coherence as well as population component. Here, by renormalizing all prefactors and substituting density matrix $\rho_{e_m e_n}(t)$ obtained by quantum master equatio, the analytical solution of the corresponding signal can be thus calculated.

A. Spectral signal I

A.1. Coherence component

For the coherence dynamic, the density matrix $\rho_{e_m e_n}(t)$ is constructed by [1]

$$
\rho_{e_m e_n}(t) = e^{-\mathbf{i}(\omega_{e_m e_n} - \mathbf{i}\gamma_{e_m e_n})t} \tag{S5}
$$

whereas $\omega_{e_m e_n}$ denotes the energy difference between electronic excited states, while $\gamma_{e_m e_n}^{-1}$ quantifies the dephasing of the vibrational coherence. The coherence component of spectral signal arises from transition pathway I is given for the following different conditions

- If $\sigma_0 < 2\gamma_{e_ke_i} - \gamma_{e_je_i}$

$$S_i^{I,co}(\omega_{\text{-}}, T_i, \Delta T) = \Re \sum_{e_i}^{3} \sum_{\substack{e_j \\ (j\neq i)}}^{3} \sum_{\substack{e_k \\ (k\neq i)}}^{3} \frac{\mathbf{i}\alpha_{e_ke_i}\alpha_{e_je_k}\rho_{e_je_i}(T_i + \frac{T_0}{2})}{-\omega_{e_je_i} + \mathbf{i}(\gamma_{e_je_i} + \frac{4}{T_0})}$$
$$\times \frac{e^{-\frac{\mathbf{i}}{2}(\omega_{\text{-}}+\omega_{+}+2\omega_{e_ke_i}+\omega_{e_je_i}-\mathbf{i}(2\gamma_{e_ke_i}+\gamma_{e_je_i}+\frac{2}{T_0}+\sigma_0))\Delta T}}{(2(\omega_{\text{-}} + \omega_{e_ke_i}) - \omega_{e_je_i} - \mathbf{i}(2\gamma_{e_ke_i} - \gamma_{e_je_i}))(2(\omega_{\text{-}} + \omega_{e_ke_i}) - \omega_{e_je_i} - \mathbf{i}(2\gamma_{e_ke_i} - \gamma_{e_je_i} + 2\sigma_0))} \tag{S6}$$

- If $2\gamma_{e_ke_i} - \gamma_{e_je_i} < \sigma_0 < 2\gamma_{e_ke_i} + \frac{2}{T_0}$

$$S_i^{I,co}(\omega_{\text{-}}, T_i, \Delta T) =$$
$$\Re \sum_{e_i}^{3} \sum_{\substack{e_j \\ (j\neq i)}}^{3} \sum_{\substack{e_k \\ (k\neq i)}}^{3} \frac{\mathbf{i}\alpha_{e_ke_i}\alpha_{e_je_k}\rho_{e_je_i}(T_i + \frac{T_0}{2})}{(-\omega_{e_je_i} + \mathbf{i}(\gamma_{e_je_i} + \frac{4}{T_0}))(2(\omega_{\text{-}} + \omega_{e_ke_i}) - \omega_{e_je_i} - \mathbf{i}(2\gamma_{e_ke_i} - \gamma_{e_je_i}))}$$
$$\times [\frac{e^{-\frac{\mathbf{i}}{2}(\omega_{\text{-}}+\omega_{+}+2\omega_{e_ke_i}+\omega_{e_je_i}-\mathbf{i}(2\gamma_{e_ke_i}+\gamma_{e_je_i}+\frac{2}{T_0}+\sigma_0))\Delta T}}{2(\omega_{\text{-}} + \omega_{e_ke_i}) - \omega_{e_je_i} - \mathbf{i}(2\gamma_{e_ke_i} - \gamma_{e_je_i} + 2\sigma_0)}$$
$$+ \frac{e^{-\frac{\mathbf{i}}{2}(\omega_{\text{-}}+\omega_{+}+2\omega_{e_ke_i}-\mathbf{i}(2\gamma_{e_ke_i}+\frac{2}{T_0}+\sigma_0))\Delta T}}{2(\omega_{\text{-}} + \omega_{e_ke_i}) - \omega_{e_je_i} - \mathbf{i}(2\gamma_{e_ke_i} - \gamma_{e_je_i} - 2\sigma_0)}] \tag{S7}$$

- If $2\gamma_{e_ke_i} - \gamma_{e_je_i} < \sigma_0 \& 2\gamma_{e_ke_i} + \frac{2}{T_0} < \sigma_0$

$$S_i^{I,co}(\omega_{\text{-}}, T_i, \Delta T) =$$
$$\Re \sum_{e_i}^{3} \sum_{\substack{e_j \\ (j\neq i)}}^{3} \sum_{\substack{e_k \\ (k\neq i)}}^{3} \frac{\mathbf{i}\alpha_{e_ke_i}\alpha_{e_je_k}\rho_{e_je_i}(T_i + \frac{T_0}{2})}{(-\omega_{e_je_i} + \mathbf{i}(\gamma_{e_je_i} + \frac{4}{T_0}))(2(\omega_{\text{-}} + \omega_{e_ke_i}) - \omega_{e_je_i} - \mathbf{i}(2\gamma_{e_ke_i} - \gamma_{e_je_i}))}$$
$$\times [\frac{e^{\frac{\mathbf{i}}{2}(\omega_{\text{-}}-\omega_{+}-2\omega_{e_ke_i}+\mathbf{i}(2\gamma_{e_ke_i}+\frac{2}{T_0}+\sigma_0))\Delta T}}{2(\omega_{\text{-}} + \omega_{e_ke_i}) - \omega_{e_je_i} - \mathbf{i}(2\gamma_{e_ke_i} - \gamma_{e_je_i} - 2\sigma_0)})$$
$$+ \frac{e^{-\frac{\mathbf{i}}{2}(\omega_{\text{-}}-\omega_{+}+2\omega_{e_ke_i}+\omega_{e_je_i}-\mathbf{i}(2\gamma_{e_ke_i}+\gamma_{e_je_i}+\frac{2}{T_0}+\sigma_0))\Delta T}}{2(\omega_{\text{-}} + \omega_{e_ke_i}) - \omega_{e_je_i} - \mathbf{i}(2\gamma_{e_ke_i} - \gamma_{e_je_i} + 2\sigma_0)}] \tag{S8}$$

A.2. Population component

For the population dynamic, it's necessary to know the expansion of the diagonal density matrix[2]

$$\rho_{e_ie_i}(t) = \sum_{p}^{3} \mathbf{O}_{ip} e^{-\lambda_p t} \mathbf{O}_{pi}^{+} \rho_{pp}(0) \tag{S9}$$

Likewise, the population component of spectral signal arises from transition pathway I is also given for the following different conditions

- If $\sigma_0 < 2\gamma_{e_k e_i} - \lambda_p$

$$S_i^{I,po}(\omega_{\text{-}}, T_i, \Delta T) = \Re \sum_{e_i}^{3} \sum_{\substack{e_k \\ (k \neq i)}}^{3} \sum_{p}^{3} \frac{|\alpha_{e_i e_k}|^2 \mathbf{O}_{ip} e^{-\lambda_p (T_i + \frac{T_0}{2})} \mathbf{O}_{pi}^{+} \rho_{ii}(0)}{\lambda_p + \frac{4}{T_0}}$$
$$\times \frac{e^{-\frac{\mathbf{i}}{2}(\omega_{\text{-}} + \omega_{+} + 2\omega_{e_k e_i} - \mathbf{i}(2\gamma_{e_k e_i} + \lambda_p + \frac{2}{T_0} + \sigma_0))\Delta T}}{(2(\omega_{\text{-}} + \omega_{e_k e_i}) - \mathbf{i}(2\gamma_{e_k e_i} - \lambda_p))(2(\omega_{\text{-}} + \omega_{e_k e_i}) - \mathbf{i}(2\gamma_{e_k e_i} - \lambda_p + 2\sigma_0))} \tag{S10}$$

- If $2\gamma_{e_k e_i} - \lambda_p < \sigma_0 < 2\gamma_{e_k e_i} + \frac{2}{T_0}$

$$S_i^{I,po}(\omega_{\text{-}}, T_i, \Delta T) = \Re \sum_{\substack{e_k \\ (k \neq i)}}^{3} \sum_{e_i}^{3} \sum_{p}^{3} \frac{|\alpha_{e_i e_k}|^2 \mathbf{O}_{ip} e^{-\lambda_p (T_i + \frac{T_0}{2})} \mathbf{O}_{pi}^{+} \rho_{ii}(0)}{(\lambda_p + \frac{4}{T_0})(2(\omega_{\text{-}} + \omega_{e_k e_i}) - \mathbf{i}(2\gamma_{e_k e_i} - \lambda_p))}$$
$$\times [\frac{e^{-\frac{\mathbf{i}}{2}(\omega_{\text{-}} + \omega_{+} + 2\omega_{e_k e_i} - \mathbf{i}(2\gamma_{e_k e_i} + \lambda_p + \frac{2}{T_0} + \sigma_0))\Delta T}}{2(\omega_{\text{-}} + \omega_{e_k e_i}) - \mathbf{i}(2\gamma_{e_k e_i} - \lambda_p + 2\sigma_0)}$$
$$+ \frac{e^{-\frac{\mathbf{i}}{2}(\omega_{\text{-}} + \omega_{+} + 2\omega_{e_k e_i} - \mathbf{i}(2\gamma_{e_k e_i} + \frac{2}{T_0} + \sigma_0))\Delta T}}{2(\omega_{\text{-}} + \omega_{e_k e_i}) - \mathbf{i}(2\gamma_{e_k e_i} - \lambda_p - 2\sigma_0)}] \tag{S11}$$

- If $2\gamma_{e_k e_i} - \lambda_p < \sigma_0 \& 2\gamma_{e_k e_i} + \frac{2}{T_0} < \sigma_0$

$$S_i^{I,po}(\omega_{\text{-}}, T_i, \Delta T) = \Re \sum_{\substack{e_k \\ (k \neq i)}}^{3} \sum_{e_i}^{3} \sum_{p}^{3} \frac{|\alpha_{e_i e_k}|^2 \mathbf{O}_{ip} e^{-\lambda_p (T_i + \frac{T_0}{2})} \mathbf{O}_{pi}^{+} \rho_{ii}(0)}{(\lambda_p + \frac{4}{T_0})(2(\omega_{\text{-}} + \omega_{e_k e_i}) - \mathbf{i}(2\gamma_{e_k e_i} - \lambda_p))}$$
$$\times [\frac{e^{\frac{\mathbf{i}}{2}(\omega_{\text{-}} - \omega_{+} - 2\omega_{e_k e_i} + \mathbf{i}(2\gamma_{e_k e_i} + \frac{2}{T_0} + \sigma_0))\Delta T}}{2(\omega_{\text{-}} + \omega_{e_k e_i}) - \mathbf{i}(2\gamma_{e_k e_i} - \lambda_p - 2\sigma_0)})$$
$$+ \frac{e^{-\frac{\mathbf{i}}{2}(\omega_{\text{-}} - \omega_{+} + 2\omega_{e_k e_i} - \mathbf{i}(2\gamma_{e_k e_i} + \lambda_p + \frac{2}{T_0} + \sigma_0))\Delta T}}{2(\omega_{\text{-}} + \omega_{e_k e_i}) - \mathbf{i}(2\gamma_{e_k e_i} - \lambda_p + 2\sigma_0)}] \tag{S12}$$

Obtaining the explicit expressions of signal, we investigate the dependence of effectively narrow bandwidth σ_0 of pump light from the SPDC process on the Q-USRS signal, as shown in Fig. S4. Broad bandwidth σ_0 is unable to resolve the peaks of spectral, which implies a decline in spectral resolution.

In addition, we investigate the dependence of entanglement time τ_0 of the nonlinear crystal on the Q-USRS signal, as shown in Fig.S5. As seen in the signal, the large τ_0 could destroy the temporal resolution of the spectral as the population, as well as coherence dynamics, vanished with respect to large τ_0. In the Fig. S5(b), as the $\frac{\tau_0}{2}$ rises larger than the timescale of the time-evolving dynamics $\rho_{e_2,e_2}(T + \frac{\tau_0}{2})$, the peaks which correspond to the transition of $|e_2\rangle \to |e_1\rangle$ and $|e_2\rangle \to |e_3\rangle$ are

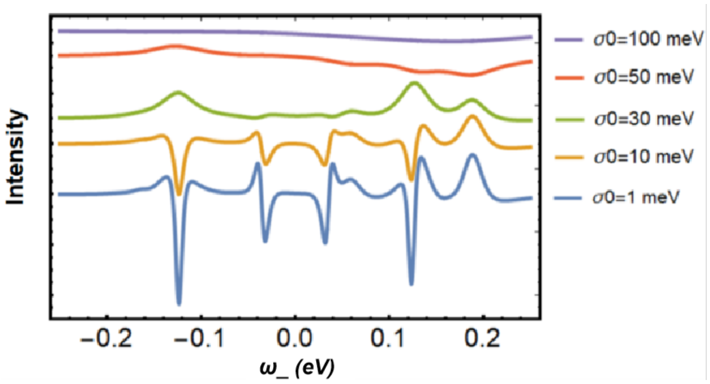

Fig. S4. Q-USRS signal with entangled photons depends on various σ_0. $\tau_0 = 25$ fs,$T = 0$ fs, $\omega_+ = 0.3$ eV; $\omega_1 = 2.25$ eV,$\omega_2 = \omega_3 = 2.1$ eV, $J = 30$ meV referring to PBI trimers used in the main text.

indeed strongly destroyed, meanwhile the oscillation information from corresponding coherence is also lost.

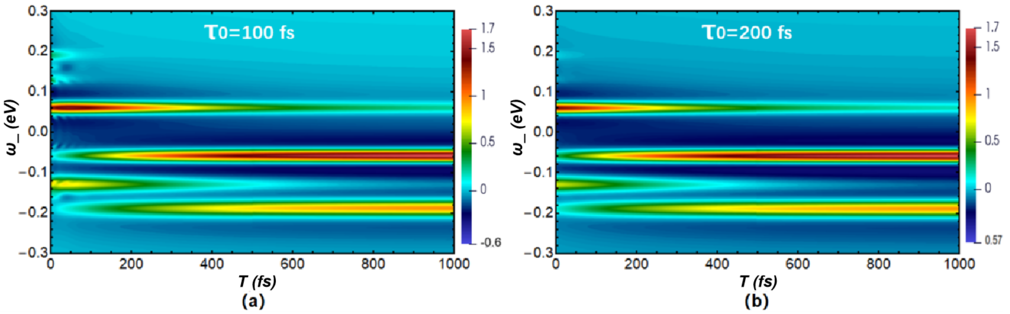

Fig. S5. (a) Q-USRS signal with entangled photons with $\tau_0 = 100$ fs; (b) Q-USRS signal with entangled photons from Eq.(7a) with $\tau_0 = 200$ fs. Herein, all other parameters $\sigma_0 = 1$ meV $\omega_+ = 0.3$ eV; $\omega_1 = 2.25$ eV, $\omega_2 = \omega_3 = 2.1$ eV, $J = 30$ meV are referring to PBI trimers used in the main text.

In order to compare the spectroscopic features induced by utilizing entangled photons, we apply a new set of PBI trimer parameters from the molecular model in Ref. [3]. As demonstrated in Fig.S6, the dynamics of coherence as well as of population various with arriving time are resolved. In general, the simulation results for these two sets of parameters of PBI trimer with different configurations share similar features in molecular dynamics, such as peaks' evolutionary information, while the difference stands in the timescale of molecular dynamics, Fig.S6 shows a longer time evolution of multi-excited states in molecules. In particular, in Fig.S6(b), coherence signals oscillate and decay faster, and the timescale also declines to very short, which means only smaller τ_0 is able to resolve the coherence dynamics. This can be achieved by tuning the parameters of the SPDC process.

B. Background signal I

As seen in Eq.(4a) in the main text, the second term is defined as a background signal, again expanding the coherence component as well as

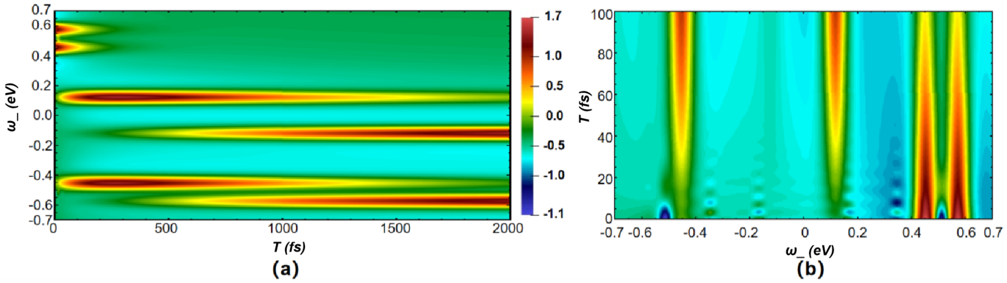

Fig. S6. (a)Q-USRS signal with entangled photons from Eq.(7a) with $\sigma_0 = 1$ meV $\tau_0 = 25$ fs, $\omega_+ = 0.3$ eV; $\omega_1 = 2.75$ eV, $\omega_2 = \omega_3 = 2.25$ eV, $J = 60$ meV referring to PBI trimers. (b) Zoom-in of (a) for a short timescale.

the population component.

B.1. Coherence component

The coherence component of the background signal arises from the transition pathway I is given for the following different conditions

• If $\sigma_0 < \gamma_{e_j e_i}$

$$
\begin{aligned}
S_{ii}^{I,co}(\omega_-, T_i, \Delta T) = \Re \sum_{e_i}^{3} \sum_{\substack{e_j \\ (j\neq i)}}^{3} \sum_{\substack{e_k \\ (k\neq i)}}^{3} \frac{\mathbf{i}\alpha_{e_k e_i}\alpha_{e_j e_k}\rho_{e_j e_i}(T_i + \frac{T_0}{2})}{-\omega_{e_j e_i} + \mathbf{i}\gamma_{e_j e_i}} \\
\times \Big[\frac{e^{-\frac{\mathbf{i}}{2}(-\omega_- + \omega_+ + \omega_{e_j e_i} - \mathbf{i}(\gamma_{e_j e_i} + \frac{2}{T_0} + \sigma_0))\Delta T}}{(\omega_{e_j e_i} - \mathbf{i}(\gamma_{e_j e_i} + \frac{4}{T_0}))(\omega_{e_j e_i} - \mathbf{i}(\gamma_{e_j e_i} - 2\sigma_0))} \\
+ \frac{e^{-\frac{\mathbf{i}}{2}(-\omega_- + \omega_+ + 2\omega_{e_j e_i} - \mathbf{i}(2\gamma_{e_j e_i} + \frac{2}{T_0} + \sigma_0))\Delta T}}{(\omega_{e_j e_i} - \mathbf{i}(\gamma_{e_j e_i} + \frac{4}{T_0}))(\omega_{e_j e_i} - \mathbf{i}(\gamma_{e_j e_i} + 2\sigma_0))} \\
- \frac{e^{-\frac{\mathbf{i}}{2}(-\omega_- + \omega_+ + 2\omega_{e_j e_i} - \mathbf{i}(2\gamma_{e_j e_i} + \frac{2}{T_0} - \sigma_0))\Delta T}}{(\omega_{e_j e_i} - \mathbf{i}(\gamma_{e_j e_i} + \frac{4}{T_0}))(\omega_{e_j e_i} - \mathbf{i}(\gamma_{e_j e_i} - 2\sigma_0))}\Big]
\end{aligned}
\tag{S13}
$$

• If $\sigma_0 > \gamma_{e_j e_i}$

$$
\begin{aligned}
S_{ii}^{I,co}(\omega_-, T_i, \Delta T) = \Re \sum_{e_i}^{3} \sum_{\substack{e_j \\ (j\neq i)}}^{3} \sum_{\substack{e_k \\ (k\neq i)}}^{3} \frac{\mathbf{i}\alpha_{e_k e_i}\alpha_{e_j e_k}\rho_{e_j e_i}(T_i + \frac{T_0}{2})}{-\omega_{e_j e_i} + \mathbf{i}\gamma_{e_j e_i}} \\
\times \Big[\frac{e^{-\frac{\mathbf{i}}{2}(-\omega_- + \omega_+ + \omega_{e_j e_i} - \mathbf{i}(\gamma_{e_j e_i} + \frac{2}{T_0} + \sigma_0))\Delta T}}{(\omega_{e_j e_i} - \mathbf{i}(\gamma_{e_j e_i} + \frac{4}{T_0}))(\omega_{e_j e_i} - \mathbf{i}(\gamma_{e_j e_i} - 2\sigma_0))} \\
+ \frac{e^{-\frac{\mathbf{i}}{2}(-\omega_- + \omega_+ + 2\omega_{e_j e_i} - \mathbf{i}(2\gamma_{e_j e_i} + \frac{2}{T_0} + \sigma_0))\Delta T}}{(\omega_{e_j e_i} - \mathbf{i}(\gamma_{e_j e_i} + \frac{4}{T_0}))(\omega_{e_j e_i} - \mathbf{i}(\gamma_{e_j e_i} + 2\sigma_0))}\Big]
\end{aligned}
\tag{S14}
$$

B.2. Population component

The population component of the background signal arises from the transition pathway I is given for the following different conditions

• If $\sigma_0 < \lambda_p$

$$S_{ii}^{I,po}(\omega_-, T_i, \Delta T) = \Re \sum_{\substack{e_k \\ (k\neq i)}}^{3} \sum_{e_i}^{3} \sum_{p}^{3} \frac{-|\alpha_{e_i e_k}|^2 \mathbf{O}_{ip} e^{-\lambda_p (T_i + \frac{T_0}{2})} \mathbf{O}_{pi}^{+} \rho_{ii}(0)}{\lambda_p(\lambda_p + \frac{4}{T_0})}$$
$$\times \Big(\frac{e^{-\frac{\mathbf{i}}{2}(-\omega_- + \omega_+ - \mathbf{i}(\lambda_p + \frac{2}{T_0} + \sigma_0))\Delta T}}{\lambda_p - 2\sigma_0} + \frac{e^{-\frac{\mathbf{i}}{2}(-\omega_- + \omega_+ - \mathbf{i}(2\lambda_p + \frac{2}{T_0} + \sigma_0))\Delta T}}{\lambda_p + 2\sigma_0}$$
$$- \frac{e^{-\frac{\mathbf{i}}{2}(-\omega_- + \omega_+ - \mathbf{i}(2\lambda_p + \frac{2}{T_0} - \sigma_0))\Delta T}}{\lambda_p - 2\sigma_0}\Big) \tag{S15}$$

• If $\sigma_0 > \lambda_p$

$$S_{ii}^{I,po}(\omega_-, T_i, \Delta T) = \Re \sum_{\substack{e_k \\ (k\neq i)}}^{3} \sum_{e_i}^{3} \sum_{p}^{3} \frac{-|\alpha_{e_i e_k}|^2 \mathbf{O}_{ip} e^{-\lambda_p (T_i + \frac{T_0}{2})} \mathbf{O}_{pi}^{+} \rho_{ii}(0)}{\lambda_p(\lambda_p + \frac{4}{T_0})}$$
$$\times \Big(\frac{e^{-\frac{\mathbf{i}}{2}(-\omega_- + \omega_+ - \mathbf{i}(\lambda_p + \frac{2}{T_0} + \sigma_0))\Delta T}}{\lambda_p - 2\sigma_0} + \frac{e^{-\frac{\mathbf{i}}{2}(-\omega_- + \omega_+ - \mathbf{i}(2\lambda_p + \frac{2}{T_0} + \sigma_0))\Delta T}}{\lambda_p + 2\sigma_0}\Big) \tag{S16}$$

C. Spectral signal III

Invoking the double-sided Feynman diagram as shown in Fig.S1, spectral signal or transition pathway III arises from the interchange of the interaction sequence of the two photons with the sample molecule($\omega_s \longleftrightarrow \omega_i$).

C.1. Coherence component

The coherence component of the spectral signal arises from the transition pathway III is given for the following different conditions

•If $\sigma_0 < 2(\gamma_{e_k e_i} - \gamma_{e_j e_i}) - \frac{2}{T_0} \& \sigma_0 < 2\gamma_{e_k e_i} - \gamma_{e_j e_i} \& \sigma_0 < \gamma_{e_j e_i} + \frac{2}{T_0}$

$$S_{i}^{III,co}(\omega_-, T_i, \Delta T) =$$
$$\Re \sum_{e_i}^{3} \sum_{\substack{e_j \\ (j\neq i)}}^{3} \sum_{\substack{e_k \\ (k\neq i)}}^{3} \frac{\mathbf{i}\alpha_{e_k e_i}\alpha_{e_j e_k}\rho_{e_j e_i}(T_i + \frac{T_0}{2})}{(-\omega_{e_j e_i} + \mathbf{i}(\gamma_{e_j e_i} + \frac{4}{T_0}))(2(\omega_- - \omega_{e_k e_i}) + \omega_{e_j e_i} + \mathbf{i}(2\gamma_{e_k e_i} - \gamma_{e_j e_i}))}$$
$$\times \Big[\frac{-e^{\frac{\mathbf{i}}{2}(\omega_- + \omega_+ - \omega_{e_j e_i} + \mathbf{i}(\gamma_{e_j e_i} + \frac{2}{T_0} - \sigma_0))\Delta T}}{2(\omega_- - \omega_{e_k e_i}) + \omega_{e_j e_i} + \mathbf{i}(2\gamma_{e_k e_i} - \gamma_{e_j e_i} - 2\sigma_0)}$$
$$+ \frac{e^{\frac{\mathbf{i}}{2}(\omega_- + \omega_+ - \omega_{e_j e_i} + \mathbf{i}(\gamma_{e_j e_i} + \frac{2}{T_0} + \sigma_0))\Delta T}}{2(\omega_- - \omega_{e_k e_i}) + \omega_{e_j e_i} + \mathbf{i}(2\gamma_{e_k e_i} - \gamma_{e_j e_i} + 2\sigma_0)}\Big] \tag{S17}$$

•If $\gamma_{e_j e_i} + \frac{2}{T_0} < \sigma_0 < 2\gamma_{e_k e_i} - \gamma_{e_j e_i} \& \gamma_{e_j e_i} + \frac{2}{T_0} < \sigma_0 < 2(\gamma_{e_k e_i} - \gamma_{e_j e_i}) - \frac{2}{T_0}$

or $2\gamma_{e_ke_i}-\gamma_{e_je_i}<\sigma_0<2(\gamma_{e_ke_i}-\gamma_{e_je_i})-\frac{2}{T_0}$

$$S_i^{III,co}(\omega_{\text{-}},T_i,\Delta T)=$$
$$\Re\sum_{e_i}^{3}\sum_{\substack{e_j\\(j\neq i)}}^{3}\sum_{\substack{e_k\\(k\neq i)}}^{3}\frac{\mathbf{i}\alpha_{e_ke_i}\alpha_{e_je_k}\rho_{e_je_i}(T_i+\frac{T_0}{2})}{(-\omega_{e_je_i}+\mathbf{i}(\gamma_{e_je_i}+\frac{4}{T_0}))(2(\omega_{\text{-}}-\omega_{e_ke_i})+\omega_{e_je_i}+\mathbf{i}(2\gamma_{e_ke_i}-\gamma_{e_je_i}))}$$
$$\times\frac{e^{\frac{\mathbf{i}}{2}(\omega_{\text{-}}+\omega_{+}-\omega_{e_je_i}+\mathbf{i}(\gamma_{e_je_i}+\frac{2}{T_0}+\sigma_0))\Delta T}}{2(\omega_{\text{-}}-\omega_{e_ke_i})+\omega_{e_je_i}+\mathbf{i}(2\gamma_{e_ke_i}-\gamma_{e_je_i}+2\sigma_0)} \tag{S18}$$

•If $2(\gamma_{e_ke_i}-\gamma_{e_je_i})-\frac{2}{T_0}<\sigma_0<2\gamma_{e_ke_i}-\gamma_{e_je_i}\&2(\gamma_{e_ke_i}-\gamma_{e_je_i})-\frac{2}{T_0}<\sigma_0<\gamma_{e_je_i}+\frac{2}{T_0}$

$$S_i^{III,co}(\omega_{\text{-}},T_i,\Delta T)=$$
$$\Re\sum_{e_i}^{3}\sum_{\substack{e_j\\(j\neq i)}}^{3}\sum_{\substack{e_k\\(k\neq i)}}^{3}\frac{\mathbf{i}\alpha_{e_ke_i}\alpha_{e_je_k}\rho_{e_je_i}(T_i+\frac{T_0}{2})}{(-\omega_{e_je_i}+\mathbf{i}(\gamma_{e_je_i}+\frac{4}{T_0}))(2(\omega_{\text{-}}-\omega_{e_ke_i})+\omega_{e_je_i}+\mathbf{i}(2\gamma_{e_ke_i}-\gamma_{e_je_i}))}$$
$$\times[\frac{e^{-\frac{\mathbf{i}}{2}(\omega_{\text{-}}-\omega_{+}-2(\omega_{e_ke_i}-\omega_{e_je_i})+\mathbf{i}(2(\gamma_{e_ke_i}-\gamma_{e_je_i})-\frac{2}{T_0}-\sigma_0))\Delta T}-e^{\frac{\mathbf{i}}{2}(\omega_{\text{-}}+\omega_{+}-\omega_{e_je_i}+\mathbf{i}(\gamma_{e_je_i}+\frac{2}{T_0}-\sigma_0))\Delta T}}{2(\omega_{\text{-}}-\omega_{e_ke_i})+\omega_{e_je_i}+\mathbf{i}(2\gamma_{e_ke_i}-\gamma_{e_je_i}-2\sigma_0)}$$
$$+\frac{e^{\frac{\mathbf{i}}{2}(\omega_{\text{-}}+\omega_{+}-\omega_{e_je_i}+\mathbf{i}(\gamma_{e_je_i}+\frac{2}{T_0}+\sigma_0))\Delta T}}{2(\omega_{\text{-}}-\omega_{e_ke_i})+\omega_{e_je_i}+\mathbf{i}(2\gamma_{e_ke_i}-\gamma_{e_je_i}+2\sigma_0)}] \tag{S19}$$

• If $2(\gamma_{e_ke_i}-\gamma_{e_je_i})-\frac{2}{T_0}<\sigma_0<2\gamma_{e_ke_i}-\gamma_{e_je_i}\&\gamma_{e_je_i}+\frac{2}{T_0}<\sigma_0<2\gamma_{e_ke_i}-\gamma_{e_je_i}$

or $2(\gamma_{e_ke_i}-\gamma_{e_je_i})-\frac{2}{T_0}<\sigma_0\&2\gamma_{e_ke_i}-\gamma_{e_je_i}<\sigma_0$

$$S_i^{III,co}(\omega_{\text{-}},T_i,\Delta T)=$$
$$\Re\sum_{e_i}^{3}\sum_{\substack{e_j\\(j\neq i)}}^{3}\sum_{\substack{e_k\\(k\neq i)}}^{3}\frac{\mathbf{i}\alpha_{e_ke_i}\alpha_{e_je_k}\rho_{e_je_i}(T_i+\frac{T_0}{2})}{(-\omega_{e_je_i}+\mathbf{i}(\gamma_{e_je_i}+\frac{4}{T_0}))(2(\omega_{\text{-}}-\omega_{e_ke_i})+\omega_{e_je_i}+\mathbf{i}(2\gamma_{e_ke_i}-\gamma_{e_je_i}))}$$
$$\times[\frac{e^{-\frac{\mathbf{i}}{2}(\omega_{\text{-}}-\omega_{+}-2(\omega_{e_ke_i}-\omega_{e_je_i})+\mathbf{i}(2(\gamma_{e_ke_i}-\gamma_{e_je_i})-\frac{2}{T_0}-\sigma_0))\Delta T}}{2(\omega_{\text{-}}-\omega_{e_ke_i})+\omega_{e_je_i}+\mathbf{i}(2\gamma_{e_ke_i}-\gamma_{e_je_i}-2\sigma_0)}$$
$$+\frac{e^{\frac{\mathbf{i}}{2}(\omega_{\text{-}}+\omega_{+}-\omega_{e_je_i}+\mathbf{i}(\gamma_{e_je_i}+\frac{2}{T_0}+\sigma_0))\Delta T}}{2(\omega_{\text{-}}-\omega_{e_ke_i})+\omega_{e_je_i}+\mathbf{i}(2\gamma_{e_ke_i}-\gamma_{e_je_i}+2\sigma_0)}] \tag{S20}$$

C.2. Population component

The population of the spectral signal arises from the transition pathway III is given for the following different conditions

• If $\sigma_0 < 2(\gamma_{e_ke_i} - \lambda_p) - \frac{2}{T_0} \& \sigma_0 < 2\gamma_{e_ke_i} - \lambda_p \& \sigma_0 < \lambda_p + \frac{2}{T_0}$

$$S_i^{III,po}(\omega_{\text{-}}, T_i, \Delta T) = \Re \sum_{\substack{e_k \\ (k\neq i)}}^{3} \sum_{e_i}^{3} \sum_{p}^{3} \frac{|\alpha_{e_ie_k}|^2 \mathbf{O}_{ip} e^{-\lambda_p(T_i + \frac{T_0}{2})} \mathbf{O}^+_{pi} \rho_{ii}(0)}{(\lambda_p + \frac{4}{T_0})(2(\omega_{\text{-}} - \omega_{e_ke_i}) + \mathbf{i}(2\gamma_{e_ke_i} - \lambda_p))} \times [\frac{-e^{\frac{\mathbf{i}}{2}(\omega_{\text{-}} + \omega_+ + \mathbf{i}(\lambda_p + \frac{2}{T_0} - \sigma_0))\Delta T}}{2(\omega_{\text{-}} - \omega_{e_ke_i}) + \mathbf{i}(2\gamma_{e_ke_i} - \lambda_p - 2\sigma_0)} + \frac{e^{\frac{\mathbf{i}}{2}(\omega_{\text{-}} + \omega_+ + \mathbf{i}(\lambda_p + \frac{2}{T_0} + \sigma_0))\Delta T}}{2(\omega_{\text{-}} - \omega_{e_ke_i}) + \mathbf{i}(2\gamma_{e_ke_i} - \lambda_p + 2\sigma_0)}] \tag{S21}$$

• If $\lambda_p + \frac{2}{T_0} < \sigma_0 < 2\gamma_{e_ke_i} - \lambda_p \& \lambda_p + \frac{2}{T_0} < \sigma_0 < 2(\gamma_{e_ke_i} - \lambda_p) - \frac{2}{T_0}$ or $2\gamma_{e_ke_i} - \lambda_p < \sigma_0 < 2(\gamma_{e_ke_i} - \lambda_p) - \frac{2}{T_0}$

$$S_i^{III,po}(\omega_{\text{-}}, T_i, \Delta T) = \Re \sum_{\substack{e_k \\ (k\neq i)}}^{3} \sum_{e_i}^{3} \sum_{p}^{3} \frac{|\alpha_{e_ie_k}|^2 \mathbf{O}_{ip} e^{-\lambda_p(T_i + \frac{T_0}{2})} \mathbf{O}^+_{pi} \rho_{ii}(0)}{(\lambda_p + \frac{4}{T_0})(2(\omega_{\text{-}} - \omega_{e_ke_i}) + \mathbf{i}(2\gamma_{e_ke_i} - \lambda_p))} \times \frac{e^{\frac{\mathbf{i}}{2}(\omega_{\text{-}} + \omega_+ + \mathbf{i}(\lambda_p + \frac{2}{T_0} + \sigma_0))\Delta T}}{2(\omega_{\text{-}} - \omega_{e_ke_i}) + \mathbf{i}(2\gamma_{e_ke_i} - \lambda_p + 2\sigma_0)} \tag{S22}$$

• If $2(\gamma_{e_ke_i} - \lambda_p) - \frac{2}{T_0} < \sigma_0 < 2\gamma_{e_ke_i} - \lambda_p \& 2(\gamma_{e_ke_i} - \lambda_p) - \frac{2}{T_0} < \sigma_0 < \lambda_p + \frac{2}{T_0}$

$$S_i^{III,po}(\omega_{\text{-}}, T_i, \Delta T) = \Re \sum_{\substack{e_k \\ (k\neq i)}}^{3} \sum_{e_i}^{3} \sum_{p}^{3} \frac{|\alpha_{e_ie_k}|^2 \mathbf{O}_{ip} e^{-\lambda_p(T_i + \frac{T_0}{2})} \mathbf{O}^+_{pi} \rho_{ii}(0)}{(\lambda_p + \frac{4}{T_0})(2(\omega_{\text{-}} - \omega_{e_ke_i}) + \mathbf{i}(2\gamma_{e_ke_i} - \lambda_p))} \times [\frac{e^{-\frac{\mathbf{i}}{2}(\omega_{\text{-}} - \omega_+ - 2\omega_{e_ke_i} + \mathbf{i}(2(\gamma_{e_ke_i} - \lambda_p) - \frac{2}{T_0} - \sigma_0))\Delta T} - e^{\frac{\mathbf{i}}{2}(\omega_{\text{-}} + \omega_+ + \mathbf{i}(\lambda_p + \frac{2}{T_0} - \sigma_0))\Delta T}}{2(\omega_{\text{-}} - \omega_{e_ke_i}) + \mathbf{i}(2\gamma_{e_ke_i} - \lambda_p - 2\sigma_0)} + \frac{e^{\frac{\mathbf{i}}{2}(\omega_{\text{-}} + \omega_+ + \mathbf{i}(\lambda_p + \frac{2}{T_0} + \sigma_0))\Delta T}}{2(\omega_{\text{-}} - \omega_{e_ke_i}) + \mathbf{i}(2\gamma_{e_ke_i} - \lambda_p + 2\sigma_0)}] \tag{S23}$$

• If $2(\gamma_{e_ke_i} - \lambda_p) - \frac{2}{T_0} < \sigma_0 < 2\gamma_{e_ke_i} - \lambda_p \& \lambda_p + \frac{2}{T_0} < \sigma_0 < 2\gamma_{e_ke_i} - \lambda_p$

or $2(\gamma_{e_ke_i} - \lambda_p) - \frac{2}{T_0} < \sigma_0 \& 2\gamma_{e_ke_i} - \lambda_p < \sigma_0$

$$\begin{aligned} S_i^{III,po}(\omega_{\text{-}}, T_i, \Delta T) =& \Re \sum_{\substack{e_k \\ (k\neq i)}}^{3} \sum_{e_i}^{3} \sum_{p}^{3} \frac{|\alpha_{e_ie_k}|^2 \mathbf{O}_{ip} e^{-\lambda_p (T_i + \frac{T_0}{2})} \mathbf{O}_{pi}^{+} \rho_{ii}(0)}{(\lambda_p + \frac{4}{T_0})(2(\omega_{\text{-}} - \omega_{e_ke_i}) + \mathbf{i}(2\gamma_{e_ke_i} - \lambda_p))} \\ & \times [\frac{e^{-\frac{\mathbf{i}}{2}(\omega_{\text{-}} - \omega_{+} - 2\omega_{e_ke_i} + \mathbf{i}(2(\gamma_{e_ke_i} - \lambda_p) - \frac{2}{T_0} - \sigma_0))\Delta T}}{2(\omega_{\text{-}} - \omega_{e_ke_i}) + \mathbf{i}(2\gamma_{e_ke_i} - \lambda_p - 2\sigma_0)} \\ & + \frac{e^{\frac{\mathbf{i}}{2}(\omega_{\text{-}} + \omega_{+} + \mathbf{i}(\lambda_p + \frac{2}{T_0} + \sigma_0))\Delta T}}{2(\omega_{\text{-}} - \omega_{e_ke_i}) + \mathbf{i}(2\gamma_{e_ke_i} - \lambda_p + 2\sigma_0)}] \end{aligned} \tag{S24}$$

D. Background signal III

D.1. Coherence component

The coherence of the background signal arises from the transition pathway III is given for the following different conditions

- If $\sigma_0 < \gamma_{e_je_i}$

$$\begin{aligned} S_{ii}^{III,co}(\omega_{\text{-}}, T_i, \Delta T) =& \Re \sum_{e_i}^{3} \sum_{\substack{e_j \\ (j\neq i)}}^{3} \sum_{\substack{e_k \\ (k\neq i)}}^{3} \frac{\mathbf{i}\alpha_{e_ke_i}\alpha_{e_je_k}\rho_{e_je_i}(T_i + \frac{T_0}{2})}{-\omega_{e_je_i} + \mathbf{i}\gamma_{e_je_i}} \\ & \times \frac{e^{\frac{\mathbf{i}}{2}(\omega_{\text{-}} + \omega_{+} - 2\omega_{e_je_i} + \mathbf{i}(2\gamma_{e_je_i} + \frac{2}{T_0} + \sigma_0))\Delta T}}{(\omega_{e_je_i} - \mathbf{i}(\gamma_{e_je_i} + \frac{4}{T_0}))(\omega_{e_je_i} - \mathbf{i}(\gamma_{e_je_i} + 2\sigma_0))} \end{aligned} \tag{S25}$$

- If $\sigma_0 > \gamma_{e_je_i}$

$$\begin{aligned} S_{ii}^{III,co}(\omega_{\text{-}}, T_i, \Delta T) =& \Re \sum_{e_i}^{3} \sum_{\substack{e_j \\ (j\neq i)}}^{3} \sum_{\substack{e_k \\ (k\neq i)}}^{3} \frac{\mathbf{i}\alpha_{e_ke_i}\alpha_{e_je_k}\rho_{e_je_i}(T_i + \frac{T_0}{2})}{-\omega_{e_je_i} + \mathbf{i}\gamma_{e_je_i}} \\ & \times [\frac{e^{\frac{\mathbf{i}}{2}(\omega_{\text{-}} + \omega_{+} - \omega_{e_je_i} + \mathbf{i}(\gamma_{e_je_i} + \frac{2}{T_0} + \sigma_0))\Delta T}}{(\omega_{e_je_i} - \mathbf{i}(\gamma_{e_je_i} + \frac{4}{T_0}))(\omega_{e_je_i} - \mathbf{i}(\gamma_{e_je_i} - 2\sigma_0))} \\ & + \frac{e^{\frac{\mathbf{i}}{2}(\omega_{\text{-}} + \omega_{+} - 2\omega_{e_je_i} + \mathbf{i}(2\gamma_{e_je_i} + \frac{2}{T_0} + \sigma_0))\Delta T}}{(\omega_{e_je_i} - \mathbf{i}(\gamma_{e_je_i} + \frac{4}{T_0}))(\omega_{e_je_i} - \mathbf{i}(\gamma_{e_je_i} + 2\sigma_0))}] \end{aligned} \tag{S26}$$

D.2. Population component

The population of the background signal arises from the transition pathway III is given for the following different conditions

• If $\sigma_0 < \lambda_p$

$$S_{ii}^{III,po}(\omega_{\text{-}}, T_i, \Delta T) = \Re \sum_{\substack{e_k \\ (k\neq i)}}^{3} \sum_{e_i}^{3} \sum_{p}^{3} \frac{-|\alpha_{e_ie_k}|^2 \mathbf{O}_{ip} e^{-\lambda_p(T_i+\frac{T_0}{2})} \mathbf{O}_{pi}^{+} \rho_{ii}(0) e^{\frac{\mathbf{i}}{2}(\omega_{\text{-}}+\omega_{+}+\mathbf{i}(2\lambda_p+\frac{2}{T_0}+\sigma_0))\Delta T}}{\lambda_p(\lambda_p+\frac{4}{T_0})(\lambda_p+2\sigma_0)} \tag{S27}$$

• If $\sigma_0 > \lambda_p$

$$S_{ii}^{III,po}(\omega_{\text{-}}, T_i, \Delta T) = \Re \sum_{\substack{e_k \\ (k\neq i)}}^{3} \sum_{e_i}^{3} \sum_{p}^{3} \frac{-|\alpha_{e_ie_k}|^2 \mathbf{O}_{ip} e^{-\lambda_p(T_i+\frac{T_0}{2})} \mathbf{O}_{pi}^{+} \rho_{ii}(0)}{\lambda_p(\lambda_p+\frac{4}{T_0})} \times \left[\frac{e^{\frac{\mathbf{i}}{2}(\omega_{\text{-}}+\omega_{+}+\mathbf{i}(\lambda_p+\frac{2}{T_0}+\sigma_0))\Delta T}}{\lambda_p-2\sigma_0} + \frac{e^{\frac{\mathbf{i}}{2}(\omega_{\text{-}}+\omega_{+}+\mathbf{i}(2\lambda_p+\frac{2}{T_0}+\sigma_0))\Delta T}}{\lambda_p+2\sigma_0}\right] \tag{S28}$$

4. Q-USRS COINCIDENCE COUNTING SIGNAL WITH UNCORRELATED PHOTONS

Since there exists no more correlation between twin photons, such that the two-photon wavefunction is separated

$$\Phi(\omega_s, \omega_i) = A(\omega_s - \omega_{s0})A(\omega_i - \omega_{i0}) = \frac{\sigma_0^2}{(\omega_s - \omega_{s0})^2 + \sigma_0^2} \frac{\sigma_0^2}{(\omega_i - \omega_{i0})^2 + \sigma_0^2} \tag{S29}$$

herein ω_{s0} and ω_{i0} denote as the center frequency of photon pulse, while σ_0 defines the spectral width of photon pulses. By replacing the two-photon wavefunction in Eq.6 in the main text as well as in Eq.S3, we are able to calculate the coincidence counting signal of USRS with uncorrelated photons. Nonetheless, we investigate $T_i = T_s = T$, and set $\omega_{s0} = \omega_{i0} = 0$ here.

Following the former logic of calculation, we split the spectral signal into the coherence component as well as the population component.

A. Spectral signal I

A.1. Coherence component

$$S_i^{I,co}(\omega_{\text{-}}, T, \Delta T) = \Re \sum_{e_i}^{3} \sum_{\substack{e_j \\ (j\neq i)}}^{3} \sum_{\substack{e_k \\ (k\neq i)}}^{3} \frac{\alpha_{e_ke_i}\alpha_{e_je_k}\rho_{e_je_i}(T)}{(-\omega_{e_je_i} + \mathbf{i}(\gamma_{e_je_i} + 4\sigma_0))(\omega_{\text{-}} + \omega_{e_ke_i} - \mathbf{i}(2\gamma_{e_ke_i} + 2\sigma_0))} \tag{S30}$$

A.2. Population component

$$S_i^{I,po}(\omega_{\text{-}},T,\Delta T)=\Re\sum_{\substack{e_k\\(k\neq i)}}^{3}\sum_{e_i}^{3}\sum_{p}^{3}\frac{\mathbf{i}|\alpha_{e_ie_k}|^2\mathbf{O}_{ip}e^{-\lambda_pT}\mathbf{O}_{pi}^{+}\rho_{ii}(0)}{(\lambda_p+4\sigma_0)(\omega_{\text{-}}+\omega_{e_ke_i}-\mathbf{i}(2\gamma_{e_ke_i}+2\sigma_0))} \tag{S31}$$

B. Spectral signal III

B.1. Coherence component

The coherence component signal of the interchange of the interaction sequence of the two photons with the sample molecule ($\omega_s \longleftrightarrow \omega_i$).

$$S_i^{III,co}(\omega_{\text{-}},T,\Delta T)= \\ \Re\sum_{e_i}^{3}\sum_{\substack{e_j\\(j\neq i)}}^{3}\sum_{\substack{e_k\\(k\neq i)}}^{3}\frac{\alpha_{e_ie_k}\alpha_{e_ke_j}\rho_{e_je_i}(T)}{(\omega_{e_je_i}-\mathbf{i}(\gamma_{e_je_i}+4\sigma_0))(\omega_{\text{-}}-\omega_{e_ke_i}+\mathbf{i}(2\gamma_{e_ke_i}+2\sigma_0))} \tag{S32}$$

B.2. Population component

$$S_i^{III,po}(\omega_{\text{-}},T,\Delta T)=\Re\sum_{\substack{e_k\\(k\neq i)}}^{3}\sum_{e_i}^{3}\sum_{p}^{3}\frac{\mathbf{i}|\alpha_{e_ie_k}|^2\mathbf{O}_{ip}e^{-\lambda_pT}\mathbf{O}_{pi}^{+}\rho_{ii}(0)}{(\lambda_p+4\sigma_0)(\omega_{\text{-}}-\omega_{e_ke_i}+\mathbf{i}(2\gamma_{e_ke_i}+2\sigma_0))} \tag{S33}$$

5. FSRS COINCIDENCE COUNTING SIGNAL USING CLASSICAL PULSES

In the case of utilizing two classical pulses, one is unable to apply coincidence counting detection, thus the selection of transition pathways vanishes. This scenario is required to exploit conventional signal detection and needs to take all signals of four transition pathways into consideration. The temporal description of classical pulses can be read as

$$\varepsilon_{s/i}(t)=e^{\mathbf{i}(\omega_{s/i}+\mathbf{i}\sigma_0)t}. \tag{S34}$$

Meanwhile, for comparison, we give up grating detection and focus on the dependence of USRS signal on Raman shift frequency $\omega_s-\omega_i$. Hence, we can rewrite all expansions of the corresponding signals

$$S_I(\omega_{\text{-}},T)=\frac{|\varepsilon_\omega|^6}{\pi}\Re\int_{-\infty}^{+\infty}dt\int_{-\infty}^{t}d\tau\langle\langle\mathbf{I}|\alpha_LG(t-\tau)\alpha_L|\rho(\tau)\rangle\rangle \\ \times e^{\mathbf{i}(\omega_s+\mathbf{i}\sigma_0)(\tau-T)}e^{-\mathbf{i}(\omega_s-\mathbf{i}\sigma_0)(t-T)}e^{-\mathbf{i}(\omega_i-\mathbf{i}\sigma_0)(\tau-T)}e^{\mathbf{i}(\omega_i+\mathbf{i}\sigma_0)(t-T)} \tag{S35}$$

$$S_{II}(\omega_{\text{-}},T)=\frac{|\varepsilon_\omega|^6}{\pi}\Re\int_{-\infty}^{+\infty}dt\int_{-\infty}^{t}d\tau\langle\langle\mathbf{I}|\alpha_LG(t-\tau)\alpha_R|\rho(\tau)\rangle\rangle \\ \times e^{\mathbf{i}(\omega_s+\mathbf{i}\sigma_0)(\tau-T)}e^{-\mathbf{i}(\omega_s-\mathbf{i}\sigma_0)(t-T)}e^{-\mathbf{i}(\omega_i-\mathbf{i}\sigma_0)(\tau-T)}e^{\mathbf{i}(\omega_i+\mathbf{i}\sigma_0)(t-T)} \tag{S36}$$

$$S_{III}(\omega_{\text{-}},T) = \frac{|\varepsilon_\omega|^6}{\pi}\Re\int_{-\infty}^{+\infty}dt\int_{-\infty}^{t}d\tau\langle\langle\mathbf{I}|\alpha_L G(t-\tau)\alpha_L|\rho(\tau)\rangle\rangle \times e^{\mathbf{i}(\omega_s+\mathbf{i}\sigma_0)(t-T)}e^{-\mathbf{i}(\omega_s-\mathbf{i}\sigma_0)(\tau-T)}e^{-\mathbf{i}(\omega_i-i\sigma_0)(t-T)}e^{\mathbf{i}(\omega_i+\mathbf{i}\sigma_0)(\tau-T)} \tag{S37}$$

$$S_{IV}(\omega_{\text{-}},T) = \frac{|\varepsilon_\omega|^6}{\pi}\Re\int_{-\infty}^{+\infty}dt\int_{-\infty}^{t}d\tau\langle\langle\mathbf{I}|\alpha_L G(t-\tau)\alpha_R|\rho(\tau)\rangle\rangle \times e^{\mathbf{i}(\omega_s+\mathbf{i}\sigma_0)(t-T)}e^{-\mathbf{i}(\omega_s-\mathbf{i}\sigma_0)(\tau-T)}e^{-\mathbf{i}(\omega_i-\mathbf{i}\sigma_0)(t-T)}e^{\mathbf{i}(\omega_i+\mathbf{i}\sigma_0)(\tau-T)} \tag{S38}$$

Note, in conventional detection, the signal only comes from the second-order expansion with the product of the corresponding input pulse field.

A. Coherence component

$$\begin{aligned}
S^{co}(\omega_{\text{-}},T) =& S_I^{co}(\omega_{\text{-}},T) + S_{II}^{co}(\omega_{\text{-}},T) + S_{III}^{co}(\omega_{\text{-}},T) + S_{IV}^{co}(\omega_{\text{-}},T) \\
=& \Re\sum_{e_i}^{3}\sum_{\substack{e_j \\ (j\neq i)}}^{3}\sum_{\substack{e_k \\ (k\neq i)}}^{3}\Big[\frac{\alpha_{e_ie_k}\alpha_{e_ke_j}\rho_{e_je_i}(T)}{(\omega_{e_je_i}-\mathbf{i}(\gamma_{e_je_i}+4\sigma_0))(\omega_{\text{-}}+\omega_{e_ke_i}-\mathbf{i}(2\gamma_{e_ke_i}+2\sigma_0))} \\
&+ \frac{\alpha_{e_ke_i}\alpha_{e_je_k}\rho_{e_ie_j}(T)}{(\omega_{e_ie_j}-\mathbf{i}(\gamma_{e_ie_j}+4\sigma_0))(\omega_{\text{-}}+\omega_{e_ie_k}-\mathbf{i}(2\gamma_{e_ie_k}+2\sigma_0))} \\
&+ \frac{\alpha_{e_ie_k}\alpha_{e_ke_j}\rho_{e_je_i}(T)}{(\omega_{e_je_i}-\mathbf{i}(\gamma_{e_je_i}+4\sigma_0))(\omega_{\text{-}}-\omega_{e_ke_i}+\mathbf{i}(2\gamma_{e_ke_i}+2\sigma_0))} \\
&+ \frac{\alpha_{e_ke_i}\alpha_{e_je_k}\rho_{e_ie_j}(T)}{(\omega_{e_ie_j}-\mathbf{i}(\gamma_{e_ie_j}+4\sigma_0))(\omega_{\text{-}}-\omega_{e_ie_k}-\mathbf{i}(2\gamma_{e_ie_k}+2\sigma_0))}\Big]
\end{aligned} \tag{S39}$$

B. Population component

$$\begin{aligned}
S^{po}(\omega_{\text{-}},T) =& S_I^{po}(\omega_{\text{-}},T) + S_{II}^{po}(\omega_{\text{-}},T) + S_{III}^{po}(\omega_{\text{-}},T) + S_{IV}^{po}(\omega_{\text{-}},T) \\
=& \Re\sum_{\substack{e_k \\ (k\neq i)}}^{3}\sum_{e_i}^{3}\sum_{p}^{3}\Big[\frac{\mathbf{i}|\alpha_{e_ie_k}|^2\mathbf{O}_{ip}e^{-\lambda_pT}\mathbf{O}_{pi}^{+}\rho_{ii}(0)}{(\lambda_p+4\sigma_0)(\omega_{\text{-}}+\omega_{e_ke_i}-\mathbf{i}(2\gamma_{e_ke_i}+2\sigma_0))} \\
&+ \frac{\mathbf{i}|\alpha_{e_ke_i}|^2\mathbf{O}_{ip}e^{-\lambda_pT}\mathbf{O}_{pi}^{+}\rho_{ii}(0)}{(\lambda_p+4\sigma_0)(\omega_{\text{-}}+\omega_{e_ie_k}-\mathbf{i}(2\gamma_{e_ie_k}+2\sigma_0))} \\
&+ \frac{\mathbf{i}|\alpha_{e_ie_k}|^2\mathbf{O}_{ip}e^{-\lambda_pT}\mathbf{O}_{pi}^{+}\rho_{ii}(0)}{(\lambda_p+4\sigma_0)(\omega_{\text{-}}-\omega_{e_ke_i}+\mathbf{i}(2\gamma_{e_ke_i}+2\sigma_0))} \\
&+ \frac{\mathbf{i}|\alpha_{e_ke_i}|^2\mathbf{O}_{ip}e^{-\lambda_pT}\mathbf{O}_{pi}^{+}\rho_{ii}(0)}{(\lambda_p+4\sigma_0)(\omega_{\text{-}}-\omega_{e_ie_k}-\mathbf{i}(2\gamma_{e_ie_k}+2\sigma_0))}\Big]
\end{aligned} \tag{S40}$$

REFERENCES

1. Z. Zhang, T. Peng, X. Nie, G. S. Agarwal, and M. O. Scully, "Entangled photons enabled time-frequency-resolved coherent raman spectroscopy and applications to electronic coherences at femtosecond scale," Light. Sci. & Appl. **11**, 274 (2022).
2. D. Abramavicius, B. Palmieri, D. V. Voronine, F. Šanda, and S. Mukamel, "Coherent multidimensional optical spectroscopy of excitons in molecular aggregates; quasiparticle versus supermolecule perspectives," Chem. Rev. **109**, 2350–2408 (2009). PMID: 19432416.
3. J. Seibt, V. Dehm, F. Würthner, and V. Engel, "Absorption spectroscopy of molecular trimers," The J. Chem. Phys. **126** (2007). 164308.
